# Dielectric Spectroscopy and Optical Density Measurement for the Online Monitoring and Control of Recombinant Protein Production in Stably Transformed *Drosophila melanogaster* S2 Cells

**DOI:** 10.3390/s18030900

**Published:** 2018-03-18

**Authors:** Jan Zitzmann, Tobias Weidner, Gerrit Eichner, Denise Salzig, Peter Czermak

**Affiliations:** 1 Institute of Bioprocess Engineering and Pharmaceutical Technology, University of Applied Sciences Mittelhessen, Wiesenstrasse 14, 35390 Giessen, Germany; jan.zitzmann@lse.thm.de (J.Z.); tobias.weidner@lse.thm.de (T.W.); denise.salzig@lse.thm.de (D.S.); 2 Mathematical Institute, Justus-Liebig University of Giessen, Arndtstrasse 2, 35392 Giessen, Germany; gerrit.eichner@math.uni-giessen.de; 3 Department of Chemical Engineering, Kansas State University, 1005 Durland Hall, Manhattan, KS 66506, USA; 4 Faculty of Biology and Chemistry, Justus Liebig University Giessen, Heinrich-Buff-Ring 17, 35392 Giessen, Germany; 5 Fraunhofer Institute for Molecular Biology and Applied Ecology (IME), Bioresources Project Group, Winchesterstrasse 2, 35394 Giessen, Germany

**Keywords:** dielectric spectroscopy, impedance spectroscopy, optical density measurements, process monitoring, process control, fermentation, recombinant protein production, *Drosophila* S2

## Abstract

The production of recombinant proteins in bioreactors requires real-time process monitoring and control to increase process efficiency and to meet the requirements for a comprehensive audit trail. The combination of optical near-infrared turbidity sensors and dielectric spectroscopy provides diverse system information because different measurement principles are exploited. We used this combination of techniques to monitor and control the growth and protein production of stably transformed *Drosophila melanogaster* S2 cells expressing antimicrobial proteins. The in situ monitoring system was suitable in batch, fed-batch and perfusion modes, and was particularly useful for the online determination of cell concentration, specific growth rate (*µ*) and cell viability. These data were used to pinpoint the optimal timing of the key transitional events (induction and harvest) during batch and fed-batch cultivation, achieving a total protein yield of ~25 mg at the 1-L scale. During cultivation in perfusion mode, the OD_880_ signal was used to control the bleed line in order to maintain a constant cell concentration of 5 × 10^7^ cells/mL, thus establishing a turbidostat/permittistat culture. With this setup, a five-fold increase in productivity was achieved and 130 mg of protein was recovered after 2 days of induced perfusion. Our results demonstrate that both sensors are suitable for advanced monitoring and integration into online control strategies.

## 1. Introduction

Sophisticated control strategies are needed to run bioprocesses within a specified operational window and to ensure system stability [1]. Typically this includes the measurement and control of physicochemical parameters such as temperature, pH, dissolved oxygen, pressure and stirrer speed. However, particularly for the production of high-value recombinant proteins, processes must also comply with comprehensive guidelines covering good manufacturing practice (GMP) and process analytical technology (PAT). Accordingly, a more detailed understanding of the process is necessary, combined with the ability to exert tighter control. This requires the online acquisition of data beyond standard parameters, especially information about cell growth and physiological status. In this context, various direct and indirect measurement principles have been evaluated and commercialized. Biomass can be quantified indirectly by off-gas analysis to measure respiration [2,3], 2D fluorescence spectroscopy to calculate the NAD(P)H content [4,5], biocalorimetry to monitor metabolic heat [6], or a combination of process data using soft sensors [7]. Direct methods include cell counting by in situ microscopy [8], near-infrared (NIR) spectroscopy [9], online optical density measurements [3,10,11,12] and dielectric spectroscopy [6,13,14,15]. Regardless of the chosen strategy, online biomass monitoring systems must meet several requirements [16]. Most important is a reliable correlation between the signal and biomass content in the reactor. The measurement principle must therefore be suitable for whichever cell type is used, e.g., it must accommodate morphology or potential adherence to growth surfaces. The measurement range, linearity, longevity, ease of evaluation, sampling frequency and operational costs must be appropriate. Furthermore the signal should not be highly susceptible to interference from factors such as gas bubbles or suspended solids. In terms of fulfilling these requirements, all competitive technologies have several distinct advantages and drawbacks, and it is beneficial to use a combination of different systems to maximize the information output [16,17].

Here we demonstrate the complementary use of dielectric spectroscopy and online optical density measurements. Both technologies are well established, commercially available and have already been used in industry [10,16,17,18,19,20]. Dielectric spectroscopy dates back more than 150 years and its theory has been extensively reviewed [13,21,22,23,24,25]. Briefly, an alternating electric field is used to measure the dielectric properties of a suspension as a function of the applied frequency. Suspended cells act as small spherical capacitors and the capacitance or permittivity therefore reflects the quantity of intact cells. The optical density probe provides information about the number of light-scattering particles in the reactor. Both systems have been used separately to monitor processes based on lepidopteran cell lines and the lytic baculovirus expression vector system (BEVS) [11,26,27,28], but they have not been tested comprehensively with stably transformed *Drosophila melanogaster* S2 cell lines (rS2 cells), which provide an equally powerful expression platform [29,30,31]. We carried out an in-depth analysis of the ability of both methods to predict the density of rS2 cells during cultivation. Based on a set of batch, fed-batch and perfusion processes, the sensor signals were compared to the reference measurement by flow cytometry, allowing a statistical analysis of sensitivity and reproducibility. The impact of cell viability on the sensor signals was evaluated in a controlled environment as well as during a real cultivation, and the sensors were used to coordinate the critical steps (induction and harvest) during batch and fed-batch cultivation. Finally, a control strategy for an intensified perfusion process based on OD_880_ readings was established in order to increase target protein yields.

## 2. Materials and Methods

### 2.1. NIR Turbidity Sensor ExCell 230 and Dielectric Spectroscopy with the Incyte Sensor

**Figure 1:**
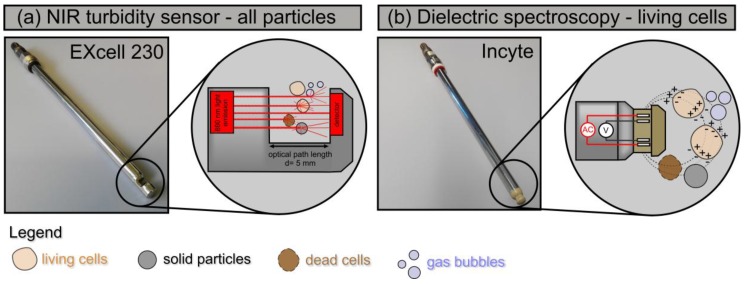
The 12-mm sensors tested in this investigation. (**a**) The EXcell 230 NIR turbidity sensor and the corresponding measurement principle of light scattering. (**b**) The Incyte dielectric spectroscopy probe with corresponding measurement principle of bio-impedance (measurement principles adapted from [29]).

We compared the NIR absorbance sensor EXcell 230 (EXNER Process Equipment, Ettlingen, Germany) and the dielectric spectroscopy system Incyte (Hamilton, Bonaduz, Switzerland). Both probes fit standard 12-mm ports, which facilitates their integration into common bioreactors. The EXcell 230 sensor is based on the scattering of NIR light at 880 nm. When transmitted through a 5-mm slit, the light is scattered by all types of suspended particles resulting in a proportional loss of intensity that can be measured (Figure 1a). Interactions with dissolved, colored media ingredients are excluded by the use of NIR light, and the signal therefore represents all particulate matter in the reactor. In contrast, the Incyte System exploits the exclusive ability of living cells to store electrical charge when exposed to an alternating electrical field at radio frequencies (Figure 1b). The Incyte system was operated at 17 distinct frequencies between 300 and 10,000 kHz (f.scan mode) allowing the construction of cell suspension beta dispersion curves. The difference in permittivity between 1000 and 10,000 kHz was used as the biomass signal (ε), whereas the complete spectrum was used to compute the characteristic frequency (f_c_), the Cole-Cole Alpha (α) and the maximal permittivity difference (Δε). The biomass signal therefore provides information about intact cells and is less influenced by other particulate matter, air bubbles or cell debris. Additionally, the Incyte system simultaneously determines conductivity at 300 kHz (κ).

### 2.2. Experimental Equipment for Cell Cultivation

#### 2.2.1. Cell Culture and Strain Maintenance

We used recombinant monoclonal *D. melanogaster* S2 cell lines expressing either gloverin from the greater wax moth *Galleria mellonella* (GmGlv) or BR021 from the harlequin ladybird *Harmonia axyridis* [30,32,33]. The genes encoding both antimicrobial peptides were controlled by the *D. melanogaster* metallothionein promoter. The constructs included a secretion signal and a V5/His_6_ or V5/GFP tag. Stable monoclonal cell lines were prepared as previously described [29,30]. The cells were grown in suspension at 27 °C in ExCell 420 serum-free medium (Sigma Aldrich, Munich, Germany) supplemented with 8–10 mM L-glutamine (Biochrom, Berlin, Germany). Selection was achieved by adding 10 µg/mL Blasticidin S or 300 µg/mL Hygromycin B (Invivogen, Toulouse, France) during subculture, depending on which selection marker was used. For maintenance, cultures were split every 3–4 days to obtain 1.5 × 10^6^ cells/mL.

#### 2.2.2. Bioreactor Setup

**Figure 2:**
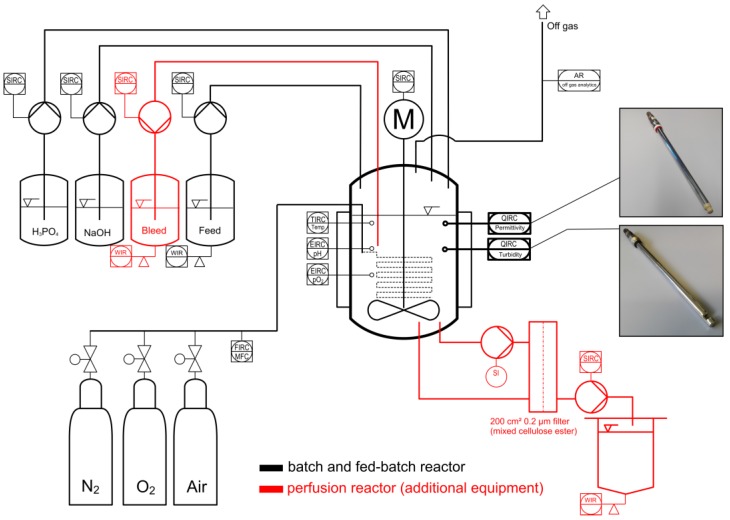
Schematic illustration of the cultivation system for batch and fed-batch culture (black symbols) and additional equipment for perfusion culture (red symbols). The sensors were directly integrated in the cultivation vessel via 12-mm ports.

The experimental setup is shown in Figure 2. The system consisted of a 2-L Labfors bioreactor with a 1-L working volume (Infors HT, Bottmingen, Switzerland) equipped with a pitched-blade impeller (3 × 45°, d = 65 mm, *n* = 70–150 rpm), a PT100 temperature probe and sensors for pH and dissolved oxygen (both from Hamilton). During cultivation, the oxygen saturation was maintained above 40% using a bubble-free aeration system combined with head space aeration, both provided via a gas mix of air, oxygen and nitrogen. The pH was maintained at 6.4 by adding 1 M sodium hydroxide or 1 M phosphoric acid. The target inoculation cell density was 1.5 × 10^6^ cells/mL. After inoculation, we carried out processes in batch, fed-batch or perfusion mode. For batch cultivation, the cells were cultivated until mid-exponential phase, before induction with 600 µM copper sulfate. The fermentation broth was harvested when cell growth started to decline. The accurate timing of both events was guided by online sensor signals. For fed-batch cultivation, a gravimetrically controlled feed line was installed to deliver glutamine-supplemented ExCell 420. The exponential feed rate was calculated to meet a target growth rate of 0.015–0.02 h^−1^ as described elsewhere [34]. The cells were induced in mid-exponential phase and the broth was harvested when the cells stopped growing, as for the batch cultivation. For the perfusion culture, an external loop was driven by a magnetically levitated centrifugal pump (PuraLev^®^ i30SU, Levitronix, Zurich, Switzerland) to minimize shear forces [35]. The bypass flow velocity was set to 100 mL/min and cell separation was achieved with a changeable 200 cm² tangential flow filter module (Microgon MiniKros mixed cellulose ester filter, 0.2 µm; Spectrum Labs, Breda, The Netherlands). Permeate flow was controlled at one reactor volume per day (0.7 mL/min) using an intermittent operating peristaltic pump (Watson Marlow 300, Rommerskirchen, Germany). The feed and bleed lines were operated with high-precision peristaltic pumps (Ismatec IPC, Cole-Parmer GmbH, Wertheim, Germany) to maintain a constant reaction volume of 1 L. The bleed line was programmed to respond to the signal of the optical sensor, thus maintaining a set point value of OD_880_ = 0.78 arbitrary units (AU). The permeate, feed and bleed flows were monitored gravimetrically using balances (Mettler Toledo, Giessen, Germany). All additional perfusion equipment was controlled by an in-house process control system based on LabVision v2.10 (HiTec Zang, Herzogenrath, Germany). Perfusion was carried out as a multistep process involving an initial batch culture step followed by a perfusion step without bleeding to reach the high target cell density, and cultivation without induction to test the online control strategy. Finally, the feed and the reactor were induced with 600 µM copper sulfate to initiate continuous protein production.

#### 2.2.3. Parallel Measurement of Turbidity and Permittivity in a Controlled Model Environment

In addition to the bioreactor experiments, turbidity and permittivity were measured in parallel using a small-scale assay and cell suspensions with adjusted viabilities. S2-GmGlv-V5/His D7 cells were grown in shake flasks to obtain a highly viable cell stock, which was split into two equal pools. One group of cells was treated with 30% ethanol for 10 s, and the other group remained untreated. The cells were centrifuged (10 min, 200× *g*) to remove the spent medium and ethanol, and were re-suspended in fresh medium. This was necessary to ensure that the signal was only affected by the proportion of dead cells and not by other changes in medium composition. Finally, both pools were mixed in different proportions and the permittivity and optical density were measured. The total cell number in all test tubes was constant at 1.3 × 10^7^ cells/mL.

#### 2.2.4. Offline Process Analytics

During cultivation, the offline cell concentration and viability were determined using a Guava easyCyte HT flow cytometer (Merck Millipore, Darmstadt, Germany). Living and dead cells were distinguished by staining with 5 mg/L propidium iodide (Carl Roth, Karlsruhe, Germany). In addition, cell morphology and viability were examined by phase contrast microscopy using a Leica DMi1 instrument (Leica Microsystems GmbH, Wetzlar, Germany) and trypan blue staining (Carl Roth). Glucose and lactate levels were measured using an enzymatic amperiometric analyzer according to the manufacturer’s instructions (Biosen C, EKF Diagnostics, Barleben, Germany). The recombinant proteins were quantified by reducing SDS-PAGE on CriterionXT 4%–20% polyacrylamide gradient gels (BioRad, Munich, Germany) as previously described [30]. Clarified culture supernatants were analyzed along with purified protein standards to enable absolute quantification. The identity of the expressed proteins was verified by their molecular weight and by western blotting using antibodies specific for the V5 (Thermo Fisher Scientific, Darmstadt, Germany) or His_6_ (Qiagen, Hilden, Germany) tags. The proteins were transferred to TransBlot Trubo PVDF membranes (Biorad), blocked with 5% bovine serum albumin (BSA) in phosphate-buffered saline (PBS), washed with 0.1% Tween-20 in PBS, and stained with a horseradish peroxidase (HRP) antibody conjugate (diluted 1:5000 in PBS with 0.05% Tween 20). The signal was detected with Clarity Western enhanced chemiluminescence substrate using the ChemiDoc system (BioRad).

### 2.3. Data Analysis

#### 2.3.1. Calibration

The sensor output and corresponding calibration were analyzed using the statistics software *R* v3.4.2 [36]. Data obtained from 11 (OD_880_) or 13 (ε) cultivations were used to establish valid equations for cell density prediction. For retrospective cases, when data representing a single cultivation were used to reconstruct a detailed growth curve for a particular experiment, we used ordinary least squares regression (OLS). Permittivity data were fitted using a simple linear regression model (Equation (1)):

(1) $$y=\beta_{0}+\beta_{1}\times x+e\;with\;e\sim N\left(0,\sigma^{2}\right)$$

The nonlinear response of the optical density sensor was modeled with a second order polynomial (Equation (2)). In both equations, y and x are the dependent and independent variables, respectively, whereas the β values represent the regression coefficients and e is the statistical noise. Based on whether the cell concentration was assigned to x or y, we distinguished between classical calibration (x = cell concentration, y = sensor signal) and inverse calibration (y = cell concentration, x = sensor signal) [37,38]:

(2) $$y=\beta_{0}+\beta_{1}\times x+\beta_{2}\times x^{2}+e\;with\;e\sim N\left(0,\sigma^{2}\right)$$

In order to access a more general model, we merged the cultivation data to yield a hierarchically structured dataset that consisted of 11 or 13 subgroups each representing the time course of a single cultivation. To account for the resulting dependencies, we used linear mixed effects (LME) models with “cultivation run” as the grouping factor [39]. Here, the response y is modeled as being composed of a systematic fixed effect (a common relationship between cell concentration and OD_880_ or ε) and a random effect that accounts for an unknown random deviation associated with each distinct experiment. Furthermore, heteroscedasticity of the residuals was considered by employing suitable variance functions. For the permittivity sensor, this results in a simple LME model (Equation (3)). Here, $y_{i,j}$ and $x_{i,j}$ refer to the *j*-th data point of the *i*-th cultivation. The fixed effects are represented in the β values, whereas $b_{1,i}$ denotes a cultivation-dependent random effect and $e_{i,j}$ are the corresponding errors:

(3) $$y_{i,j}=\beta_{0}+\left(\beta_{1}+b_{1,i}\right)\times x_{i,j}+e_{i,j}$$

For the optical density sensor, we expanded the LME model with a quadratic term, introducing an additional β value and a second random effects term, $b_{2,i\;}$ (Equation (4)):

(4) $$y_{i,j}=\beta_{0}+\left(\beta_{1}+b_{1,i}\right)\times x_{i,j}+(\beta_{2}+b_{2,i})\times x_{i,j}{}^{2}+e_{i,j}$$

As a general model for cell density prediction, which is applicable to future cultivations, we extracted the fixed effects terms because they represent systematic relationships. A more comprehensive description of the statistical assessment, including diagnostic plots as well as detailed information on employed models and their assumptions, can be found in Supplementary Material S1.

#### 2.3.2. Calculation of the Specific Growth Rate

The specific growth rate μ was defined as the constant of proportionality in the exponential growth law (Equation (5)), where N(t) refers to the absolute cell number, *t* to the elapsed time and *N*_0_ to the cell number at the beginning of the considered phase. N(t) is linked to the cell concentration X(t) via the reaction volume V(t). Equation (5) was fitted to data captured during cultivation in order to calculate the averaged specific growth rate μ for a corresponding growth phase:

(5) $$N\left(t\right)=N_{0}\times e^{\mu \times t}\;with\;N\left(t\right)=X\left(t\right)\times V\left(t\right)$$

Additionally, we tested two algorithms for the prediction of μ based on the online sensor signals. The first one uses a moving window with the process data of the past 10 h to calculate μ as slope of the linear Ln (N(t)) function. The second approximates the time course of N(t) for a 24-h interval prior to the sampling point with a polynomial of degree two [40,41] (Equation (6)). Based on the corresponding regression coefficients $a_{1–2}$ that are calculated for each time point, the growth rate can be calculated according to Equation (7):

(6) $$\frac{dN}{dt}=N\times \mu \;with\;N_{24h\;backward}=a_{2}\times t^{2}+a_{1}\times t+a_{0}$$

(7) $$\mu =\frac{dN}{N\times dt}=\frac{2\times a_{2}\times t+a_{1}}{a_{2}\times t^{2}+a_{1}\times t+a_{0}}$$

## 3. Results

### 3.1. Correlation between Cell Density, Turbidity and Permittivity during Cultivation

#### 3.1.1. Retrospective Modeling and Predictive Capabilities

Calibration is generally required to relate a parameter of interest (e.g., cell density) to the corresponding sensor output. 

**Figure 3:**
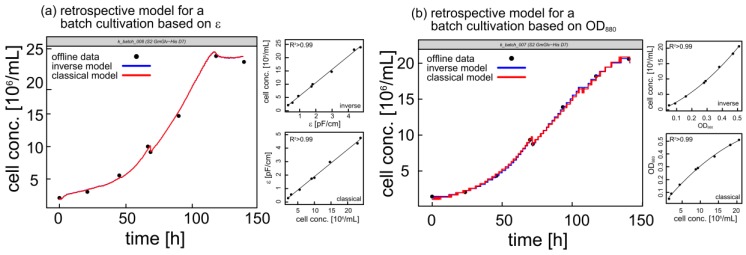
Offline cell density (black dots) and corresponding growth curves based on inverse (blue line) and classical (red line) calibrations for two representative batch cultivations. Smaller panels show corresponding calibration curves. (**a**) Permittivity-based retrospective models for cultivation k_batch_008. (**b**) OD_880_-based retrospective models for cultivation k_batch_007. The kinks at ~70 h represent induction with 600 µM copper sulfate.

For the EXcell 230 sensor, we used the dimensionless optical density in AU. The Incyte system provided a more extensive output comprising five parameters: ε, Δε, f_c_, α and κ. Out of these, ε appeared most suitable for calibration as shown by a scatter plot matrix of all variables (Supplementary Material S1). For single experiments, retrospective models were used to fill the gaps between discrete offline measurements. Thus, the monitoring of either OD_880_ or ε allowed a detailed reconstruction of the complete growth curve at the conclusion of each experiment (Figure 3). Because retrospective models are difficult to generalize, LME models were used to find common calibration functions that were valid for different cultivations with diverse process conditions (Table 1). 

**Figure 4:**
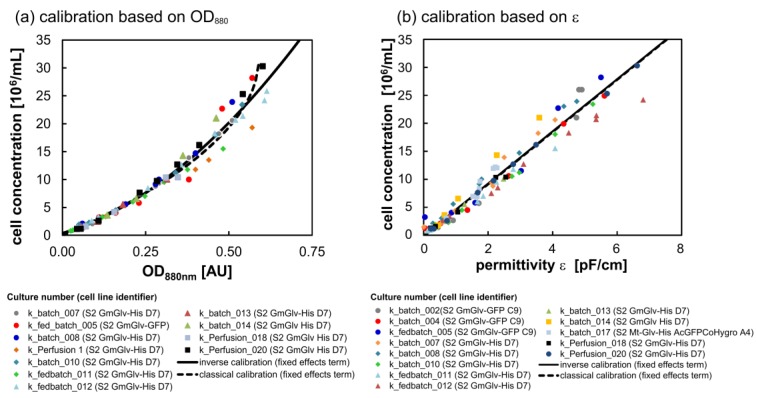
Calibration using the fixed effects terms of the classical and inverse LME models. (**a**) Scatter plot of measured viable cell concentration and the signal of the EXcell 230 NIR turbidity sensor based on exponentially growing populations. (**b**) Correlation between measured viable cell concentration and the Incyte permittivity ε (1000–10,000 kHz) based on exponentially growing populations.

**Figure A1:**
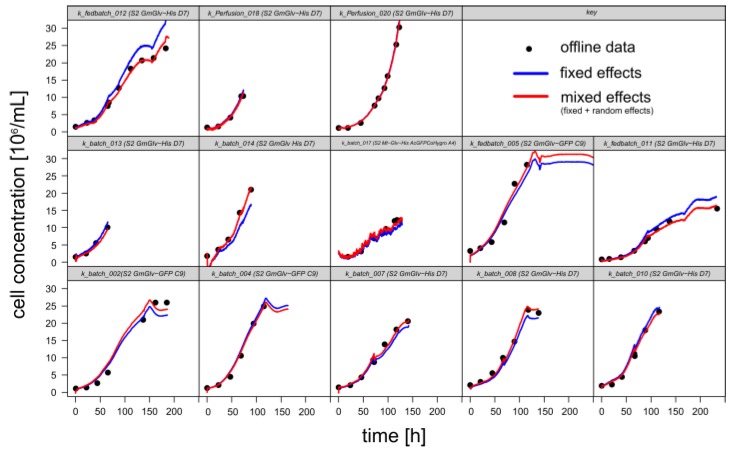
Estimated time courses of 13 cultivations based on the classical calibration of the Incyte system with a LME model. The fixed effects predictions (blue line) represent the systematic relationship, whereas the mixed effects predictions (red line) consider systematic and cultivation dependent random effects.

**Figure A2:**
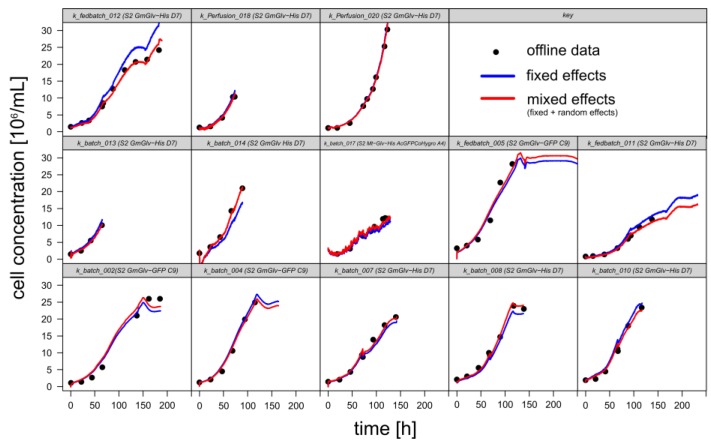
Estimated time courses of 13 cultivations based on the inverse calibration of the Incyte system with a LME model. The fixed effects predictions (blue line) represent the systematic relationship, whereas the mixed effects predictions (red line) consider systematic and cultivation dependent random effects.

**Figure A3:**
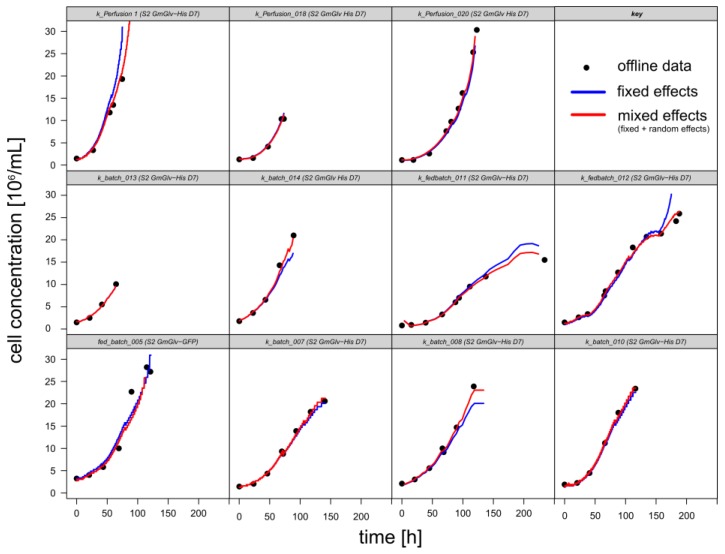
Estimated time courses of 11 cultivations based on the classical calibration of the EXcell 230 system with a LME model. The fixed effects predictions (blue line) represent the systematic relationship, whereas the mixed effects predictions (red line) consider systematic and cultivation dependent random effects.

**Figure A4:**
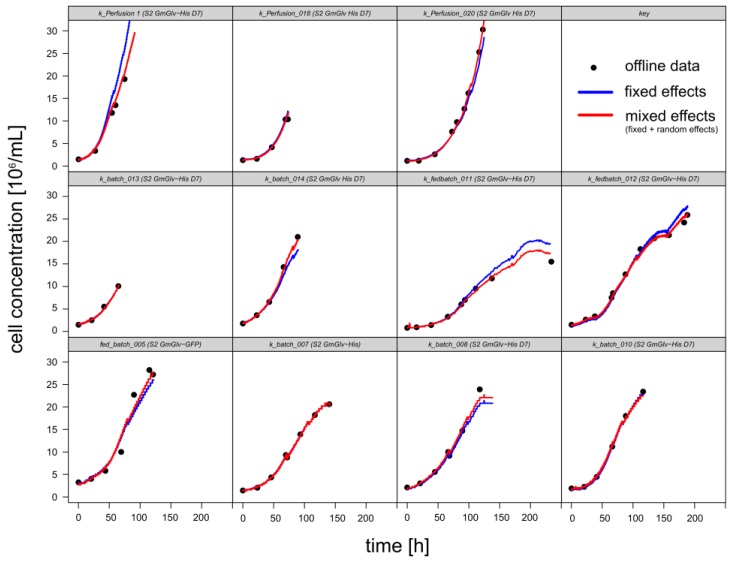
Estimated time courses of 11 cultivations based on the inverse calibration of the EXcell 230 system with a LME model. The fixed effects predictions (blue line) represent the systematic relationship, whereas the mixed effects predictions (red line) consider systematic and cultivation dependent random effects.

OD_880_-based calibration revealed high conformity between cultivations for cell densities below 10 × 10^6^ cells/mL (Figure 4a). However, deviations expand as linearity ceases at higher cell densities. Nonlinearity can be explained by the fact that, at high cell density, neighboring cells cause shading which in turn results in a non-equal contribution to the scattering signal. Another consequence is that classical and inverse calibration yield slightly different predictions at the upper end of the calibration range. In contrast, the scatter plot based on ε shows an entirely linear relationship, but also a stronger variability among different cultivations (Figure 4b). Here, inverse and classical calibrations are virtually indistinguishable. Predictions based on the four established models were in good agreement with the offline measurements, confirming that all proposed methods yield practically applicable calibration functions (Figure A1, Figure A2, Figure A3 and Figure A4).

#### 3.1.2. Timing of Induction and Harvest for Batch and Fed-Batch Cultures with High Viability

**Figure 5:**
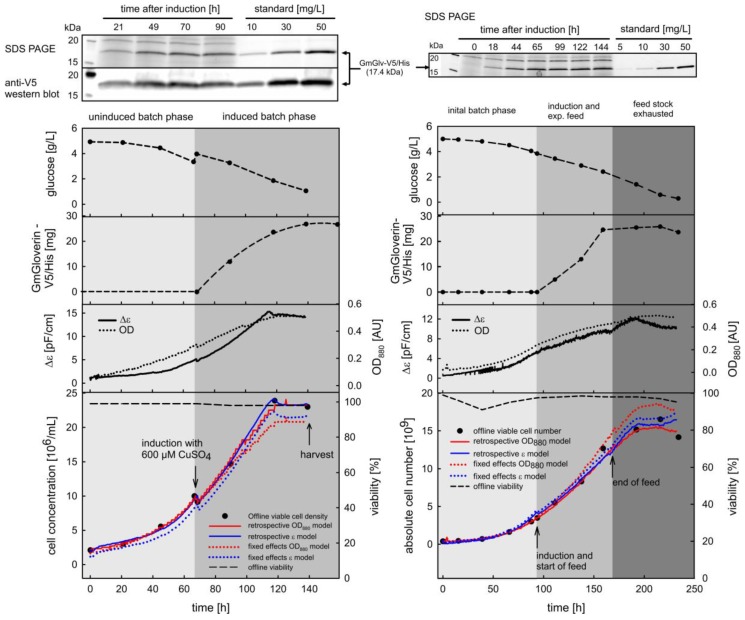
Growth curves and corresponding process data for representative batch (left panel, batch_008) and fed-batch (right panel, fed_batch_011) processes for the production of GmGlv in the cell line S2 GmGlv-His-D7. The lower panel shows the inverse LME (dotted) and inverse retrospective (solid lines) models for biomass estimation based on OD_880_ (red) or ε (blue). Note that the last point of the GmGlv concentration in the batch culture originates from the small amount of culture broth that was further cultivated after harvest.

The dynamic measurement of cell concentration was analyzed in detail for two processes in batch and fed-batch mode, where standard parameters such as dissolved oxygen, pH and temperature were kept constant to ensure optimal production conditions. Monitoring of cell growth was enabled by the inverse fixed effects models and compared to the retrospective models (Figure 5). 

**Figure 6:**
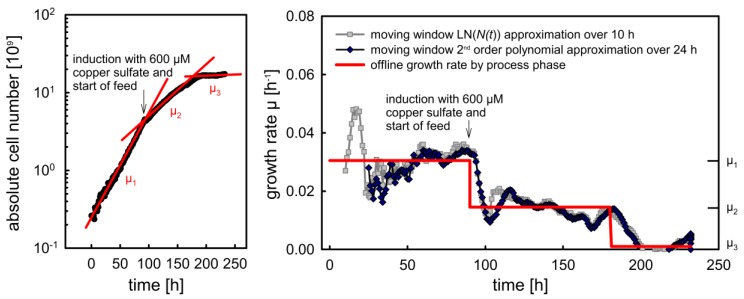
Growth rate calculation based on of cell numbers that were predicted with the inverse fixed effects model and the ε data for cultivation fed_batch_011. Offline calculation of averaged growth rates according to three different process phases (left panel and red line in the right panel), moving window-based calculations (right panel, gray and dark blue lines).

As expected, the retrospective models performed slightly better because they were exclusively related to the experiment. However, the fixed effects term of the LME model also delivered reasonable predictions and showed clear differentiation between the process phases. During the initial non-induced batch phases, cells were highly viable and the average growth rate was 0.024–0.030 h^−1^, which is in good agreement with previous reports [31]. The post-induction growth rates dropped due to the cytotoxicity of the inducer, and decreased further at the onset of nutrient depletion. The moving window-based calculation of *µ* provided a high-resolution insight to the evolution of this parameter, particularly emphasizing the transition from the non-induced to the induced state and finally to a non-proliferating state (Figure 6). Despite growth arrest at the end of the cultivation, cell viability remained >95% until harvest.

**Figure A5:**
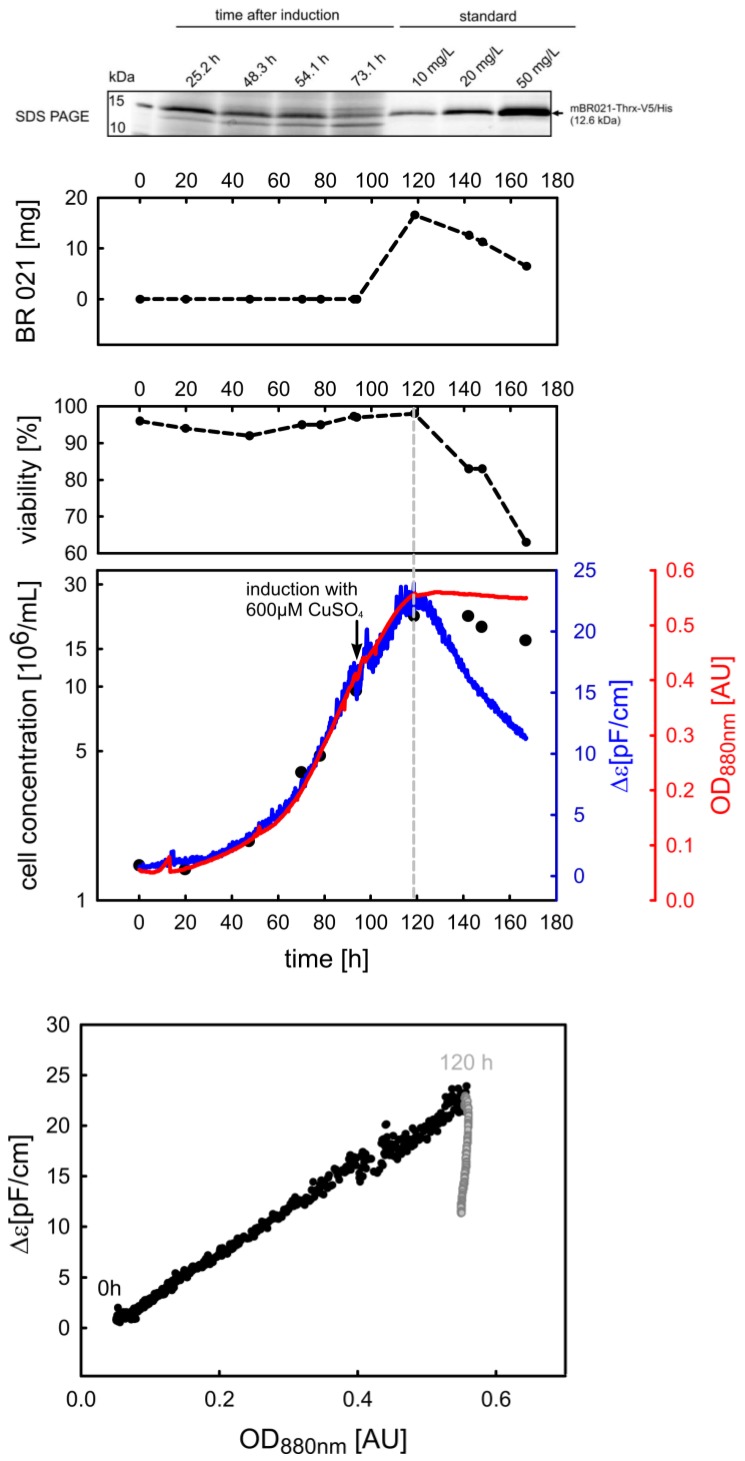
Growth and viability curves for prolonged batch cultivations for the production of BR021 in the cell line S2 Mt-BR021-V5/His E3. After entering the stationary phase and the depletion of essential nutrients, the cell population lost viability. The transition from an active to a decaying culture is visible in the corresponding Δε-OD_880_ phase trajectories. Black dots represent the process before 120 h, followed by gray dots for the subsequent time points. SDS PAGE shows that the lower cell viability is associated with protein degradation and the release of host cell protein. The shifted band position results from the exceptional high pI of 10.72 that alters the migration properties of BR021 during SDS PAGE.

As well as the prediction of the growth curve, it was necessary to relate the sensor signals to the key transitional events (induction and harvest) to gain additional benefits from their online availability. Previous statistically designed experiments on rS2 cells found that the optimal induction cell density was 7–10 × 10^6^ cells/mL [30]. We therefore used the online signals to achieve induction within this window. Thereafter, the concentration of GmGlv in the supernatant was monitored to determine the optimal window for harvest. The highest concentration of GmGlv (25 mg/L) was observed at the onset of the stationary phase. However, prolonged stationary cultivation provided no additional benefit and carries the risk of product degradation or the release of contaminating host cell proteins [30] (compare also Figure A5). Such negative effects were avoided by tight online monitoring, allowing harvesting to be initiated before cellular decay. The measurement of glucose as an alternative parameter was not suitable for this purpose because cell growth declined before the main carbon and energy source was exhausted. The early decline in cell growth may reflect either prolonged exposure to the cytotoxic inducer or the depletion of another growth-limiting substrate. In summary, dielectric spectroscopy and the online measurement of optical density were both suitable for the control of batch and fed-batch processes.

### 3.2. Viability Assessment via the Parallel Measurement of Turbidity and Permittivity

#### 3.2.1. Determining Cell Viability in a Controlled Environment

**Figure 7:**
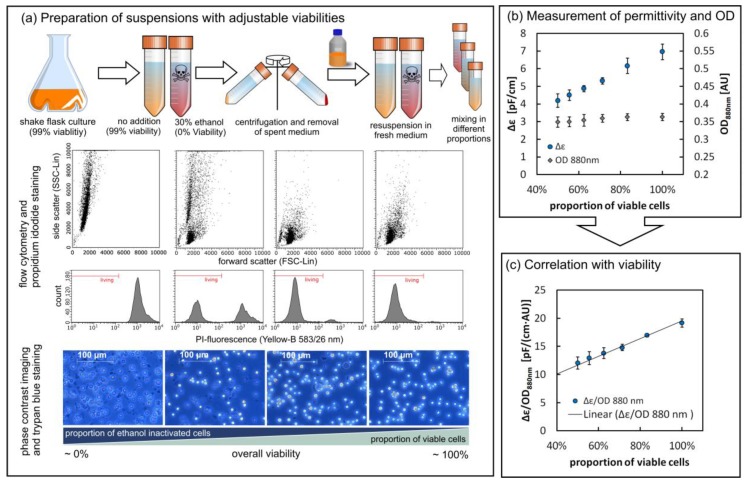
Viability assessment via the parallel measurement of turbidity and permittivity in a controlled environment. (**a**) Preparation of cell suspensions with different viabilities and analysis of the resulting cell pools via flow cytometry and microscopy. (**b**) Raw signals of Δε and OD_880nm_ show the different responses of the sensors (*n* = 4, mean ± SD). (**c**) The ratio of the two signals shows linear dependence on the viability of the cell suspension (*n* = 4, mean ± SD).

In order to investigate the influence of dead cells on the sensor outputs, we worked with standardized rS2 cell suspensions that were independent from changes in medium composition and cell aging, both of which occur in a real cultivation setting. The standardized rS2 cell suspensions allowed us to prepare mixtures in which the cell viability could be defined precisely. First we verified our assay using standard offline methods (Figure 7a). Trypan blue staining showed that the ethanol-treated cells remained macroscopically intact, but lost their ability to exclude the stain. Propidium iodide staining and flow cytometry confirmed these results and also revealed morphological changes in the light-scattering properties of dead cells, which were smaller and more granular than living cells. However after mixing both pools (99% viable, 100% dead) in defined proportions, the OD_880_ signal showed no reaction to the variations (Figure 7b). This is because the total quantity of light-scattering particles (living cells, dead cells and cell debris) was constant and apparently the changed scattering properties did not influence the overall signal. In contrast, the permittivity value dropped with the increasing proportion of defective cells, reflecting the fact that cells with leaking membranes lose their ability to store an electrical charge (Figure 7b). We also observed changes in the characteristic frequency f_c_, which may reflect differences in the cell size distribution, the specific membrane capacitance, and intracellular conductivity (Supplementary Material S2). Because the permittivity at 1000 kHz (ε) is strongly affected by a changing f_c_ value, we used the less susceptible Δε value for further analysis. Taking the quotient of Δε and OD revealed a linear dependence on viability (Figure 7c). Consequently, a stain-free viability assessment was possible via the parallel measurement of turbidity and permittivity.

#### 3.2.2. Case Study of Viability Assessment during Batch and Fed Batch Cultivation

**Figure 8:**
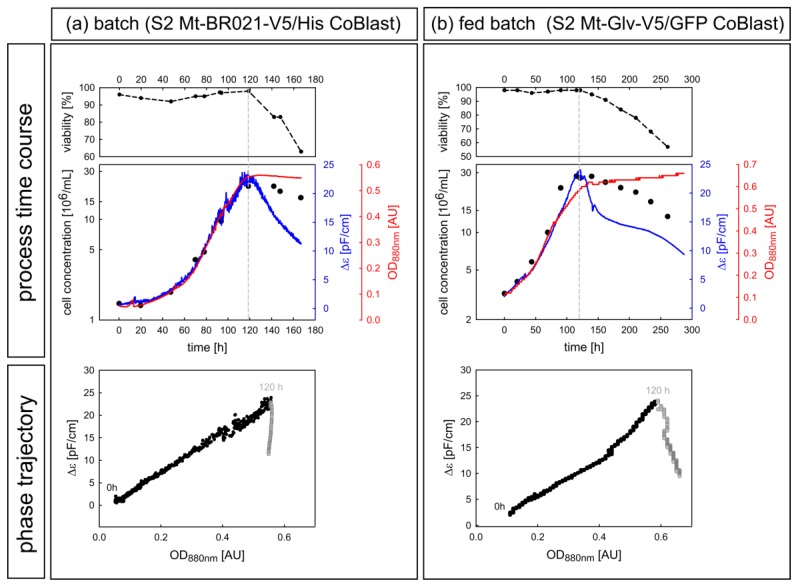
Growth and offline viability curves for prolonged batch (**a**) and fed-batch (**b**) cultivations for the two different cell lines S2 Mt-BR021-V5/His (k_batch_016) and S2 Mt-Glv-V5/GFP (k_fed_batch_005). Induction commenced at 92 h and 120 h, respectively. After entering the stationary phase and the depletion of essential nutrients, the cell population lost viability. The transition from active to decaying culture is visible in the corresponding Δε-OD_880_ phase trajectories. Black dots represent the process before 120 h, followed by gray dots for subsequent time points (lower panel).

Having demonstrated proof of concept, we used prolonged batch and fed-batch cultivations to verify the online measurement of viability under practical conditions. For both processes, cells reached their peak density at ~120 h with >95% viability, followed by progressive cell death after the depletion of essential nutrients in the stationary phase (Figure 8, upper panel). In accordance with the small-scale assay, the latter event was associated with a drop in Δε, whereas the OD_880_ remained constant over time. The transition from active to stationary culture was especially observable in the corresponding Δε-OD_880_ phase trajectories (Figure 8, lower panel), where the growth-related correlation between the two signals disappeared suddenly following the onset of growth arrest and cell death. However, because of the changing conditions and process-to-process variations, it was not possible to determine the exact percentage of viability based on the calibration with standardized rS2 cells. Nonetheless, the phase trajectory shows a sharp kink, which can be used to optimize the harvesting time. As soon as the kink appears, the degradation and contamination of our target peptide BR021 was observed, reflecting the release of proteases and host cell protein from damaged cells (Figure A5). In this context, the parallel measurement of turbidity and permittivity is suitable for online process control and allows a rapid response to detrimental events.

#### 3.2.3. Phase Trajectories of ε and Δε as Alternative Sources of Process Information

**Figure 9:**
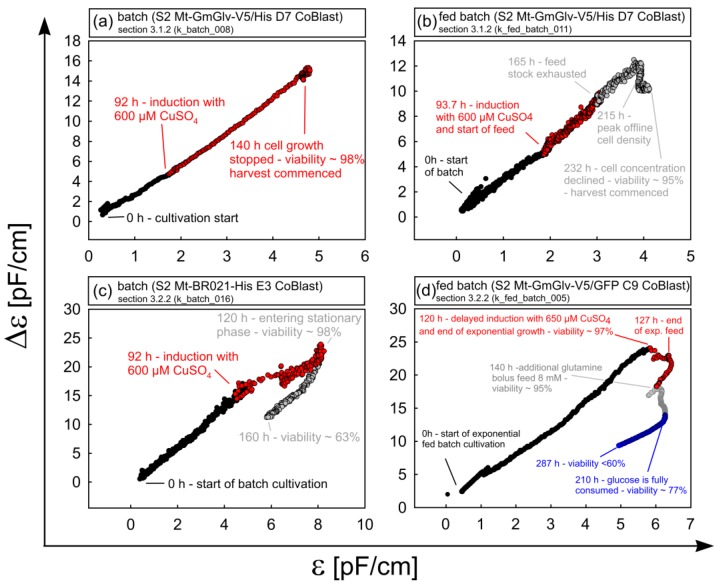
Analysis of the representative cultivations described in Section 3.1.2 (batch_008, fed_batch_011) and 3.2.2 (batch_016, fed_batch_005) by plotting the ε-Δε phase trajectories. Healthy and exponentially growing batch (**a**) and fed-batch (**b**) cultures show a predominantly linear relationship between ε and Δε. Prolonged cultures with nutrient depletion (late stage **b**–**d**) show altered trajectories reflecting physiological changes in the cell population. Different process phases and key transition points are highlighted.

As stated in Section 3.2.1, the accumulation of dead cells leads to alterations in the shape of the β-dispersion and consequently ε and Δε differed in their responses to a decaying culture. Therefore, we evaluated the practicability of ε-Δε phase trajectories as an alternative method for online viability monitoring. Figure 9 shows the ε-Δε plots corresponding to the cultivations discussed above. Where high cell viability was combined with an optimal harvest time, there was an almost linear correlation between ε and Δε (see Section 3.1.2 and Figure 9a). This applied to the majority of our cultivations. For prolonged batch and fed-batch cultivations, deviations from linearity were observed at later process sages, because of the changing cell physiology and culture conditions, and the increasing prevalence of cell death (see Section 3.2.2 and Figure 9b–d). However, these transitions were not exclusively related to viability and even occurred before massive cell death was detected. Using the fed-batch cultivation in Figure 9d as an example, the changes of direction can be related to distinct events such as the end of the exponential growth phase, feed stock depletion and complete glucose exhaustion. These events may be related to changes in intracellular conductivity and membrane capacitance. In summary, the results confirm the applicability of ε-Δε plots beyond simple viability monitoring, but also emphasize the need for comprehensive offline monitoring to assign direction changes to corresponding events.

### 3.3. OD_880_-Controlled Perfusion to Achieve a Turbidostat/Permittistat Culture with Enhanced Productivity

**Figure 10:**
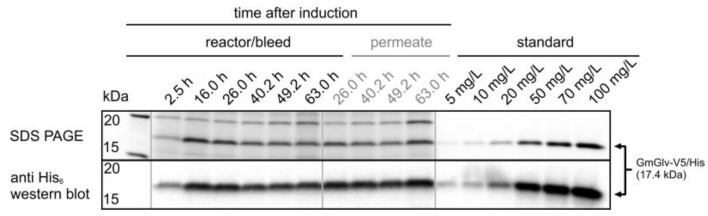
SDS-PAGE and western blot analysis of the reactor/bleed line and the perfusion stream after induction in the steady state (phase IV).

**Figure 11:**
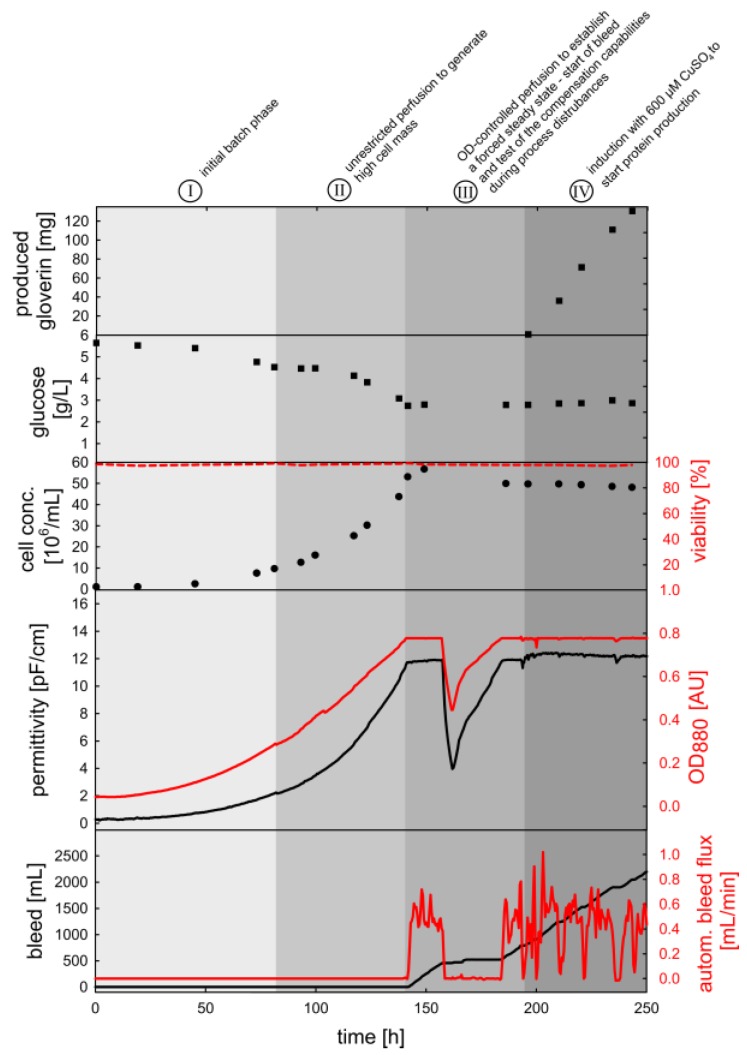
Time course of the key process variables during the four distinct phases of the perfusion cultivation of S2-GmGlv-His D7 cells. The small bleed volume increase at ~130 h is attributed to a manual manipulation on the bleed line and was not included in the bleed rate calculation.

**Figure 12:**
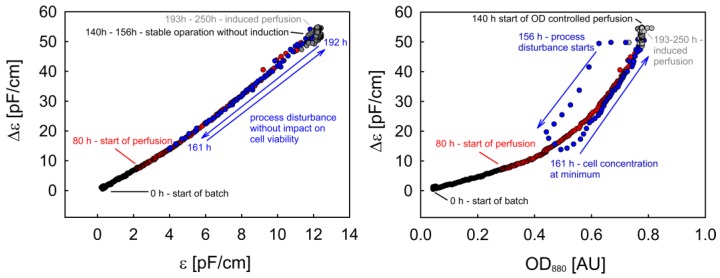
The ε-Δε and OD-Δε phase trajectories for the perfusion process.

The total protein yield in batch and fed-batch cultures is limited by finite cell growth and protein degradation during the stationary phase. Therefore, perfusion processes offer an alternative route to achieve superior productivity without growth restriction. Based on tangential flow filtration (TFF) we realized a constant nutrient supply and accordingly high cell concentrations using essentially the same equipment used in the other process modes. In order to characterize our system and to determine the effect of elevated cell concentration on productivity, the corresponding cultivation was divided into four phases (Figure 10, Figure 11 and Figure 12). The steps for generation of a high cell concentration (I, II) were followed by a test of the OD_880_-based bleed control (III) and finally induction with 600 µM copper sulfate for protein production (IV).

During the initial batch and unlimited perfusion phases (I, II), the cells proliferated exponentially with a specific growth rate of 0.029 h^−1^. Accordingly, the main carbon and energy source (glucose) was consumed, but no lactate production was observed. The target cell density of 50 × 10^6^ cells/mL was reached after 141 h. At this point, the controller started the bleed pump and the OD_880_ was kept constant at 0.78 AU for 16 h (III). Following the establishment of a forced steady state, we tested the robustness of the system. As a model disturbance, the reactor was completely filled with additional medium and half of the cells were removed afterwards to restore the original volume. Subsequently, the imbalanced system was left undisturbed, to allow for compensation by the control algorithm. Within the next 22 h, the reactor reached its previous steady state in terms of cell density and glucose concentration, demonstrating the stability of the system. Copper sulfate was then added to initiate the production of GmGlv, which was monitored offline by SDS-PAGE and anti-His_6_ western blotting (Figure 10). 

After an initial ramp-up, the concentration of GmGlv in the permeate and bleed flow stabilized with small deviations (Figure 10). In summary, this resulted in a linear increase in the total amount of recombinant protein with a final titer of 130 mg after 49 h (Figure 11). This is equivalent to a volumetric productivity of 63 mg/(L·d). Compared to the final yield of typical batch cultivations, the short continuous production phase already achieved a five-fold increase. In terms of cell density control, we observed minor fluctuations during the induced phase, which were quickly compensated by the automatically adjusted bleed rate. Overall, the OD_880_, ε and Δε were characterized by similar time courses, which is in accordance with the high cell viability (>97%) throughout cultivation and additionally confirms that both sensors can be used interchangeably for the control of a healthy turbidostat/permittistat process. The entirely linear ε-Δε phase trajectory supports the observation that the cells remained in good condition during all phases (Figure 12). 

The OD-Δε trajectory is curved due to the nonlinearity of the ExCell sensor at high cell densities. Furthermore, the rapid process disturbance is visible as a turn in the OD-Δε trajectory (Figure 12). However, this is a consequence of the slow response of the Incyte System rather than changes in cell physiology. Finally, both plots show that the system stabilizes at its previous state.

## 4. Discussion and Conclusions

### 4.1. Characterization of the ExCell 230 and the InCyte Sensors

The calibration of the ExCell 230 and InCyte sensors confirmed their suitability for the monitoring of rS2 cells. Highly accurate retrospective models (R^2^ > 0.99) allowed the fundamental analysis of growth kinetics, in agreement with previous results [30]. By combining LME models with the data from different cultivations of rS2 cells producing GmGlv, we improved the calibration procedure and developed a more general model. The resulting equations can be used for robust cell density predictions in future experiments. Our analysis showed that the inverse and classical calibration approaches are comparable. From a strict mathematical point of view, only the classical approaches ensure the correct assignment of dependent and independent variables. However, in terms of practical applications, the inverse models performed equally well and are more straightforward to calculate, especially for higher-order polynomials. This is particularly relevant for the ExCell 230 sensor, because its signal is not linearly related to cell concentration. Comprehensive evaluation of different online OD sensors in mammalian cell bioreactors showed that nonlinearity is a common property of OD sensors [10]. This is attributed to shading effects, which become more prevalent at higher cell concentrations, and similar phenomena were observed in our experiments. The use of second or third order polynomials is therefore frequently reported for the inverse calibration of OD sensors [11,26,41,42,43] and linear calibration is only conceivable at low cell concentrations [12,44]. Emerging nonlinearity was also associated with a with a stronger signal variability among the cultivations (heteroscedasticity), which in turn resulted in a lower certainty of prediction at the end of the cultivation, a behavior also observed for hybridoma cell lines [42]. Nonetheless, the ExCell 230 sensor was reliable at concentrations of up to 5 × 10^7^ rS2 cells/mL, which is in agreement with previous reports for other cell types [10,41]. These results highlight the fact that optical sensors require a suitable optical path length as the key parameter to adjust the linear measurement region, because only this region gives precise results. It is notable that interfering factors such as gas bubbles were excluded by our reactor setup, but are potential sources of error that must be considered in other reactor systems [17]. Color-related disturbances were easily excluded by the use of NIR light. Neither medium color changes during cultivation nor the addition of the strong blue copper sulfate had an impact on the online measurement (Supplementary Material S3).

Due to its measuring principle, the Incyte sensor was not susceptible to any of the disturbances mentioned above and additionally showed a linear response. This facilitated linear calibration using either the inverse [20,45] or classical [46] approach, as previously reported. However, despite the satisfactory prediction capability, the signals from different cultivations were less congruent compared to the OD sensor, which is reflected in a broader distribution of the data points around the ε-calibration curve. The origin of this variance is unclear, but a study with microorganisms showed that dielectric spectroscopy is a more complex method that still needs refinement. The same study also mentions that long-term stability is not guaranteed, and that a considerable drift in measurement capability is possible following multiple autoclaving cycles [44]. Dielectric spectroscopy is generally very sensitive to changes in morphology and the cellular interior, which may be an additional reason for the lack of congruence. Beyond the easily implemented linear ε-calibration discussed herein, there are also more complex calibration methods available. The first involves a rearrangement of the Cole-Cole equation combined with the determination of the cell-specific membrane capacitance per unit area and the cytoplasmic conductivity [47]. The second uses partial least squares (PLS) regression on the raw dielectric spectra [47,48]. In studies with CHO cells, all approaches (linear, Cole-Cole and PLS) achieved acceptable accuracy [47,49], although the PLS and Cole-Cole models were preferable in one case [49]. Whereas linear calibration provides easy access to dielectric spectroscopy, the considerable effort required for the more complex methods is worthwhile if one is interested in the additional parameters (e.g., intracellular conductivity in the Cole-Cole model) associated with these methods. Summing up our data, both sensors gave comparable results for monitoring viable rS2 cells, but we recommend their complementary application to gain a redundant source of information. As a future perspective, both outputs could be combined within an error-weighted soft sensor as proposed for other techniques [45,50].

### 4.2. Gaining Process Understanding by Online Monitoring of Cell Density, Specific Growth Rate and Viability

To gain added value from online monitoring, it is necessary to link cellular status information to the sensor signals, which in turn facilitates automated process control. In this context, dielectric spectroscopy and the online measurement of optical density have proven to be valuable tools for the online monitoring of various cell lines including CHO, Vero, hybridoma, HeLa, Sf9 and High Five cells [17,21]. In terms of insect cell culture, dielectric spectroscopy has been used mainly to monitor baculovirus-infected Sf9 cells, where the permittivity signals were used for the detection of infection-related cell swelling [51] and to determine the optimal timing for infection and harvest [52]. Accordingly online optical density measurements were also shown to correlate well with Sf9 cell density but the resulting models failed to represent the lysing cell population post-infection [11,26].

Based on these earlier results, we have demonstrated the successful adaption of both techniques for the online monitoring of rS2 cells [30]. Online monitoring allowed us to determine optimal windows for the key transitional events (induction and harvest), achieving optimized product titers and avoiding late-stage product degradation. In batch and fed-batch cultivation, induction commenced at the mid-exponential phase and subsequent production peaked at the end of growth. This is consistent with reports for other rS2 lines [30,53,54,55] and demonstrates that the productivity of the cells is strongly coupled to replication given that both processes require efficient protein synthesis. Accordingly, the specific growth rate *µ* is another key parameter suitable for monitoring. However, the direct calculation of *µ* from online raw signals is hampered by the susceptibility of the differentiation operation to noise [41]. With the help of two moving window-based filtering techniques, we improved this situation to achieve the detailed analysis of the corresponding time course of *µ*. Although we used a non-lethal and optimized copper sulfate concentration of 600 µM [30,56], the copper ions had an immediate impact on the growth rate indicating a strong influence on cell metabolism (e.g., by inhibiting key enzymes). Likewise, the monitoring of *µ* can be used to highlight nutrient depletion and even the *µ*-based automation of feeding is conceivable, as demonstrated before [56,57]. As long as the cells are highly viable, the outputs of both sensors can be used interchangeably for the purposes described above. However, the complementary use of optical density and permittivity not only provides redundancy for healthy populations but also enables the robust measurement of viability at later process stages, e.g., the OD_880_-ε phase trajectories indicate the onset of cell death as a sharp kink. Beyond this approach, also a more detailed online assessment of viability seems feasible given that recent studies using CHO cells introduced a soft sensor based on permittivity and optical density to model the evolution of viable cells, dead cells and cell debris during cultivation [45]. Because this soft sensor is based on several assumptions, phase trajectory plots will remain as an easy-to-implement and straightforward tool for the evaluation of multiple sensor readings. The ε-Δε plots were also suitable for process monitoring. During exponential growth, both parameters were linearly related, but deviations showed up during later process phases. These transitions were not exclusively related to viability, but can be interpreted as a result of changes in the physiological status and composition of the cell population. The analysis of two high-density CHO perfusion processes using similar plots provided evidence that the observed changes were related to cell shrinkage and membrane permeabilization coupled with the release of internal organelles [48]. A smaller number of large intact cells generally leads to a shift in the characteristic frequency towards higher values because smaller cells are “charged” more rapidly. The resulting shifted β dispersion curve in turn leads to direction changes in the ε-Δε plots. Our small-scale viability assay demonstrated that the reduced cell size in the ethanol-treated population was associated with an increased f_c_ (Supplementary Material S2). However, a one-to-one comparison between bioreactor cultivations and the small-scale assay is not possible because the real process involves a less clearly defined situation, and changes in cell physiology occur more fluently. The mode of cell death, either induced by toxic chemicals or as a result of nutrient depletion, also has a major impact on the dielectric spectrum [58]. Whereas solvents and detergents may disrupt the membrane and lead to a rapid traumatic cell death, starvation tends to cause major physiological changes before cell lysis [58]. This leads directly to the question of how to define cellular viability. It is clear that different methods of viability assessment give different results, depending on the parameters that are measured, which typically include membrane integrity (Trypan blue exclusion), metabolic activity (MTT assay) or proliferation capability (plate counts). Among the available techniques, dielectric spectroscopy tests for membrane integrity and changes in intracellular conductivity or specific membrane capacitance. Consequently, it is not a simple substitute for other methods, but can be regarded as an independent tool to provide additional information [47]. For example, we detected the impact of a glutamine bolus and of complete glucose exhaustion as direction changes in the ε-Δε plot. In CHO cell cultures, a decrease in f_c_ (and consequently changes in Δε) were attributed to a loss of intracellular conductivity caused by glutamine exhaustion [59]. Similarly, studies with the yeast *Saccharomyces cerevisiae* showed that intracellular conductivity varied among the different growth phases and in response to thermal stress, thus affecting the β-dispersion [60]. These findings emphasize the inherent ability of dielectric spectroscopy to provide information about the intracellular environment, but also show that comprehensive offline monitoring is necessary to identify the underlying phenomena. For research purposes, we therefore recommend the use of dielectric spectroscopy in combination with complementary methods such as spent medium analysis, respiration monitoring [47] or the determination of metabolic parameters such as the specific quantity of intracellular nucleotide triphosphates [61].

### 4.3. Process Intensification Using an OD_880_-Controlled Perfusion Process

The commercial production of biologicals in animal cells is usually carried out as fed-batch or perfusion processes [62]. Perfusion is particularly attractive because it offers a high maximum cell density (>5 × 10^7^ cells/mL) combined with superior volumetric productivity and long-term production cycles [63,64]. However, to be effective and robust, the corresponding processes require the appropriate reactor setup and the controlled adjustment of feed and bleed rates. Maintaining the perfusion-derived pseudo-steady-state in such bioreactors is especially challenging because high and potentially fluctuating cell densities can cause serve changes in the environmental conditions [19,65]. In this case, infrequent manual sampling provides insufficient information to characterize or to control the process. In order to overcome this sampling bottleneck, dielectric spectroscopy is already frequently used for perfusion monitoring [48,49,66]. For example, it allows the control and optimization of cell-specific perfusion rates (in nL/(cell·day)) [66] and the determination of optimal production temperatures for continuous protein production in CHO cells [67]. In another continuous process with hybridoma cells growing on a fluidized bed, the capacitance signal was used for closed-loop control of the glutamine feed rate [61]. Likewise, optical sensors enabled the establishment of hybridoma cultures with stationary cell counts (turbidostat) [68]. Accordingly, we used the ExCell 230 NIR sensor to maintain a constant cell concentration by automatic manipulation of the bleed rate (at a fixed perfusion rate). This not only allowed us to adjust the maximum cell density to the oxygenation capacity of our reactor system, but also resulted in a very stable process. Fluctuations of various magnitudes were easily compensated by the closed-loop controller, while online assessment of healthiness was enabled by the parallel measurement of permittivity. Using a short model production phase of 2 days, we increased the total amount of target protein from typically 25 mg for batch or fed-batch processes to ~130 mg. The linear production kinetics during perfusion shows that at least for this short period the production rate was constant at around 63 mg/(L·d). This is quite similar to the production of tissue-type plasminogen activator (~60 mg/(L·d)) in a permittivity-supervised CHO cell process [66]. In summary, the proposed process strategy outperformed the classical cultivation modes in terms of robustness and effectiveness, demonstrating the benefits associated with the transition from pure monitoring to closed-loop control. However, the system is not yet fully optimized and a further assessment of the cell-specific perfusion rate may improve nutrient utilization and increase cell-specific productivity. Also, long-term production has yet to be investigated, highlighting the issues of cell line stability and membrane fouling characteristics.

## Figures and Tables

**Table 1:** Summary of the extracted fixed effects models as generally valid equations for cell density prediction (valid below 30 × 10^6^ cells/mL): cell concentration [10^6^ cells/mL], OD_880_ [AU], ε [pF/cm].

**Prediction Based on Optical Density OD_880_**	
Inverse calibration	$cell\;conc_{predicted,inv}=0.365+\;18.457\times OD_{880}+\;42.404\;\times OD_{880}{}^{2}$
Classical calibration	$cell\;conc_{predicted,\;cl}=\frac{-0.0368+\sqrt{0.0368^{2}-\left(-4\times 0.000578\times \left(0.00319-OD_{880}\right)\right)}}{-2\times 0.000578}$
**Prediction Based on Permittivity ε**	
Inverse calibration	$cell\;conc_{predicted,\;inv}=-0.0755\;+\;4.660\times \varepsilon$
Classical calibration	$cell\;conc_{predicted,\;cl}=\;\frac{\varepsilon -0.0397}{0.214\;}$

## Supplementary Materials

The following are available online at http://www.mdpi.com/1424-8220/18/3/900/s1, File S1: Statistical analysis of the relationship between cell concentration, optical density and permittivity, File S2: Raw values of the measured parameters in the small-scale viability assay, File S3: Color change of the medium following the addition of copper sulfate.

## Abbreviations

The following abbreviations are used in this manuscript:

**Symbol**	**Name**	**Unit**
a	Regression coefficients for the approximation of N(t)	
b	Random effects coefficients	
β	Fixed effects coefficients	
BEVS	Baculovirus expression vector system	
BR021	*Harmonia axyridis* antimicrobial peptide BR021	
BSA	Bovine serum albumin	
CHO	Chinese hamster ovary cells	
e	error	
GmGlv	*Galleria mellonella* antimicrobial peptide gloverin	
HeLa	Henrietta Lacks cell line	
High Five	Ovarian cell line from the cabbage looper *Trichoplusia ni*	
MTT	3-(4,5-dimethylthiazol-2-yl)-2,5-diphenyltetrazolium bromide	
NIR	Near infrared	
OD_880_	Optical density at 880 nm	
PBS	Phosphate-buffered saline	
PVDF	Polyvinylidene difluoride	
rS2	Recombinant *Drosophila melanogaster* Schneider 2 cells	
SD	Standard deviation	
SDS PAGE	Sodium dodecylsulfate polyacrylamide gel electrophoresis	
Sf9	Clonal isolate of *Spodoptera frugiperda* Sf21 cells	
TFF	Tangential flow filtration	
Vero	African green monkey kidney epithelial cells (from verda reno = green kidney)	
f_c_	Characteristic frequency	[kHz]
N	Absolute cell number	[-]
t	Time	[h]
X	Cell concentration	[10^6^/mL]
κ	Conductivity	[mS/cm]
*µ*	Specific growth rate	[h^-1^]
Δε	Maximal permittivity difference	[pF/cm]
ε	Permittivity	[pF/cm]
