# Supplementary material for: Dielectric Spectroscopy and Optical Density Measurement for the Online Monitoring and Control of Recombinant Protein Production in Stably Transformed Drosophila melanogaster S2 Cells
Source: Sensors (Basel). 2018 Mar 18;18(3):900. doi: 10.3390/s18030900 (PMC5876727; doi:10.3390/s18030900)
Supplement: Supplementary file 1 [file sensors-18-00900-s001.zip › Supplementary S1.pdf]

sensors

MDPI

Supplementary material S1

Statistical analysis of the relationship between cell concentration (CC), optical density (OD_{880}) and permittivity (ε)

1. Basic assumptions and considerations

1.1 classical and inverse regression

The main goal of the analysis was the establishment of a valid calibration for the prediction of the cell concentration based on the measurement of optical density (OD_{880}) or permittivity (ε). In the following paragraph, we describe the fundamental ideas of classical and inverse regression [1–3] and their application to our data.

In the classical regression approach (denoted as: cl), the dependent variable y (i.e. OD_{880} or ε) is regressed on the independent variable x (i.e. cell concentration). Here, the derived signal output is to be explained by its underlying cause (i.e. higher cell concentration causes higher light scattering). The cell concentration is assumed to be free of measurement error, whereas the signal output represents a stochastic variable. In other words, the signal output can be explained as a function of cell concentration plus an unexplained error e [1], resulting from the uncertainty of the sensor measurement (Equations 1 and 2). In order to enable prediction of future cell concentrations, the model has to be rearranged to obtain the inverted classical calibration model.

$$OD = f(cell\ conc) + e_{cl} \quad (1)$$
$$\varepsilon = f(cell\ conc) + e_{cl} \quad (2)$$

Instead of rearranging the classical model, a functional relationship can also be obtained by direct regression of the cell concentration on the signal output (Equations 3 and 4).

$$Cell\ conc = f(OD) + e_{inv} \quad (3)$$
$$Cell\ conc = f(\varepsilon) + e_{inv} \quad (4)$$

This procedure is called inverse regression (denoted as: inv) and its model can be used directly to predict the cell concentration for future sensor outputs. However, this model is fit without considering which of the variables incorporates errors and which one is random, independent and error-free. Although this violates basic assumptions underlying linear regression and results in a biased model, it was mathematically shown that inverse models perform as well or even better in terms of predicting future observations [1,2]. It is obvious that classical and inverse models are not interchangeable, but as the determination coefficient tends to become 1, the difference between both will be minimized [1].

In the following analysis both methods were used for the purpose of online cell density monitoring. Thereby we focus rather on practical aspects, than giving a detailed mathematical comparison, which can be found elsewhere [1–3]. The analysis was restricted to standard diagnostic plots for regression.

[1] In statistics the errors are usually represented by ε. Because ε is preassigned to the permittivity value, we changed the designation of the errors to e

1.2 Considerations on meassurements and data aquesition

All variables in our dataset (*cell conc*, ε, OD_{880}) were determined experimentally and may contain a certain error, a situation which is usually not reflected in simple regression models. Because OD_{880} and ε values are the result of a device-internal running average calculation (by default 10 seconds for OD_{880} or 6 minutes for ε), we used these values directly without any modification. The corresponding values of cell concentration were determined by flow cytometry. In order to validate our reference method we analyzed the cell concentration of eight samples with different cell concentrations multiple times and constructed corresponding box blots (Fig. S1). Based on the plot we can conclude that the accuracy of our reference method is very high and the error of cell concentration determination can be neglected in the classical calibration procedure, where we used the mean of *cell concentration* as our independent variable. Additionally, according to Levene's test we do not reject the hypothesis of variance homogeneity (homoscedasticity) between the different samples (Output S2), indicating that our reference method seems to work over a wide range of cell concentrations.

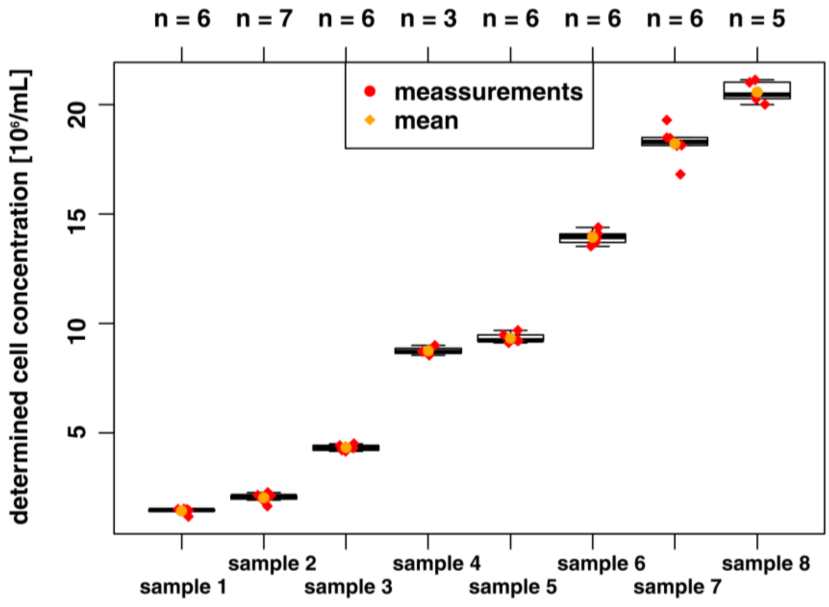

Figure S1. Box plots for eight samples with different cell concentrations analyzed multiple times by flow cytometry. Jittered raw measurement values (red) and sample means (orange) are superimposed.

```
Levene's Test for Homogeneity of Variance (center = median)
      Df F value Pr(>F)
group  7  2.0867 0.0696 .
      37
---
Signif. codes:  0 '***' 0.001 '**' 0.01 '*' 0.05 '.' 0.1 ' ' 1
```

Output S2. Results of Levene's Test for homogeneity of variance.

1.3 Further restrictions

To achieve a homogeneous dataset without data points with high leverage the analysis was restricted to cell concentrations below $35 \cdot 10^6$ cells/mL.

1.4 Software

Analysis was conducted with R v3.4.2 [4] using the following additional packages: readr [5], car [6], investr [7], GGally [8], lattice [9], lattice extra[10] and nlme [11,12].

2. Assessment of the optical density OD_{880} for the prediction of the cell concentration

2.1 Retrospective modeling with ordinary least squares (OLS) linear regression

Retrospective modeling refers to the case, where it was investigated how the data of one single experiment can be used to reconstruct a detailed growth curve. Based on an exemplary single cultivation (k_batch_007) we fitted the quadratic classical and inverse models to obtain the corresponding $\hat{\beta}$ values (Equations 5 and 6).

$$cell\ conc = \beta_{0\ inv} + \beta_{1\ inv} \cdot OD_{880} + \beta_{2\ inv} \cdot {OD_{880}}^2 + e_{inv} \qquad with\ \ e_{inv} \sim \mathcal{N}\left(0, {\sigma_{e_{inv}}}^2\right) \tag{5}$$

$$OD_{880} = \beta_{0\ cl} + \beta_{1\ cl} \cdot cell\ conc + \beta_{2\ cl} \cdot cell\ conc^2 + e_{cl} \qquad with\ \ e_{cl} \sim \mathcal{N}\left(0, {\sigma_{e_{cl}}}^2\right) \tag{6}$$

Diagnostic plots (Fig. S3) are acceptable concerning normality and homoscedasticity of the residuals. However, due to the fact that they are constructed based on only eight data points, interpretation remains difficult.

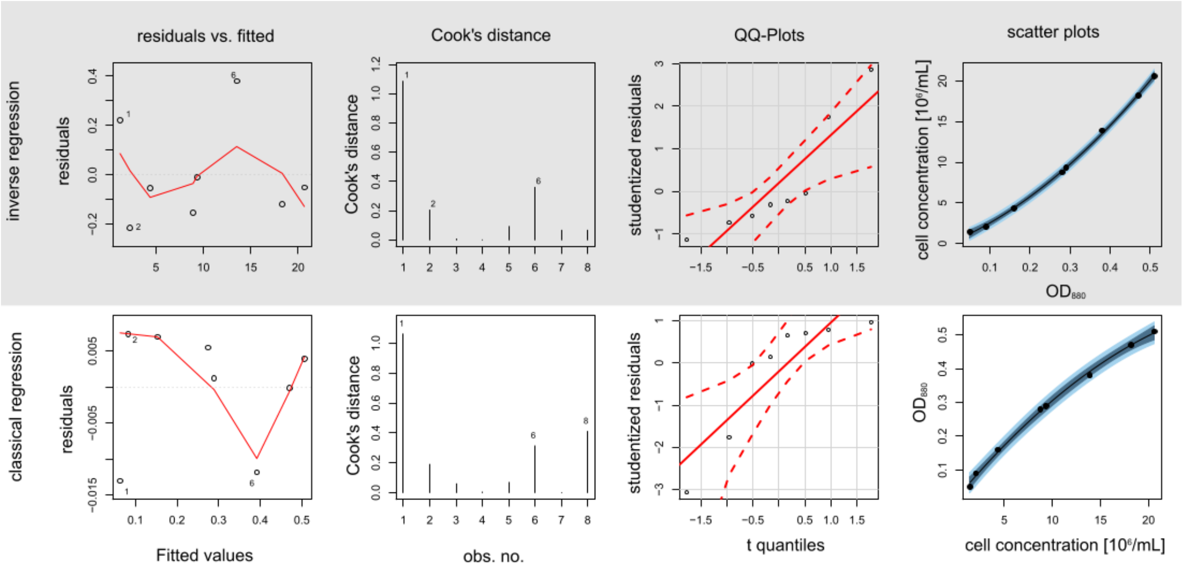

Figure S3. Diagnostic plots for inverse and classical regression: (a) Residuals vs. fitted values for homoscedasticity evaluation, (b) Cook's distance plot for determination of points with high influence, (c) QQ-Plots for normality assessment and (d) scatter plots with pointwise confidence and prediction bands (level=0.95).

A detailed summary of the fits is given in the corresponding R-outputs S4 and S5.

```
Inverse model:

Coefficients:
                         Estimate Std. Error t value Pr(>|t|)
β̂0 inv                    0.07983    0.27380   0.292 0.782334
β̂1 inv                   20.85018    2.34410   8.895 0.000299 ***
β̂2 inv                   38.20587    4.09488   9.330 0.000238 ***
---
Signif. codes:  0 '***' 0.001 '**' 0.01 '*' 0.05 '.' 0.1 ' ' 1

Residual standard error: 0.2382 on 5 degrees of freedom
Multiple R-squared:  0.9992, Adjusted R-squared:  0.9989
F-statistic:  3215 on 2 and 5 DF,  p-value: 1.683e-08
```

Output S4. Inverse regression model for cultivation k_batch_007.

```
Classical model:

Coefficients:
                          Estimate Std. Error t value Pr(>|t|)
β̂0 cl                     1.523e-02  8.862e-03   1.718   0.1463
β̂1 cl                     3.387e-02  2.095e-03  16.166 1.65e-05 ***
β̂2 cl                    -4.876e-04  9.457e-05  -5.156   0.0036 **
---
Signif. codes:  0 ‘***’ 0.001 ‘**’ 0.01 ‘*’ 0.05 ‘.’ 0.1 ‘ ’ 1

Residual standard error: 0.009577 on 5 degrees of freedom
Multiple R-squared:  0.9977, Adjusted R-squared:  0.9968
F-statistic:  1101 on 2 and 5 DF,  p-value: 2.441e-07
```

Output S5. Classical regression model for cultivation k_batch_007.

Estimations based on both models (according to Equations 7 and 9) yielded nearly identical growth curves (Fig. S6), which is particularly a result of the high determination coefficient adj. R^2. In summary, both calibration approaches can be used for retrospective modelling based on the OD_{880}.

Inverse model for k_batch_007:

$$cell\ conc_{pred} = 0.07983 + 20.85018 \cdot OD_{880} + 38.20587 \cdot {OD_{880}}^2 \qquad with\ \ adj.R^2 = 0.9989 \qquad (7)$$

Classical model for k_batch_007:

$$OD_{880} = 0.015229 + 0.033870 \cdot cell\ conc - 0.000487 \cdot cell\ conc^2 \ \ with\ \ adj.R^2 = 0.9968 \qquad (8)$$

Inverted classical model for k_batch_007:

$$cell\ conc_{pred} = \frac{-0.033870 + \sqrt{0.033870^2 - (4 \cdot 0.000487 \cdot (0.015229 - OD_{880}))}}{2\ \cdot 0.000487} \qquad (9)$$

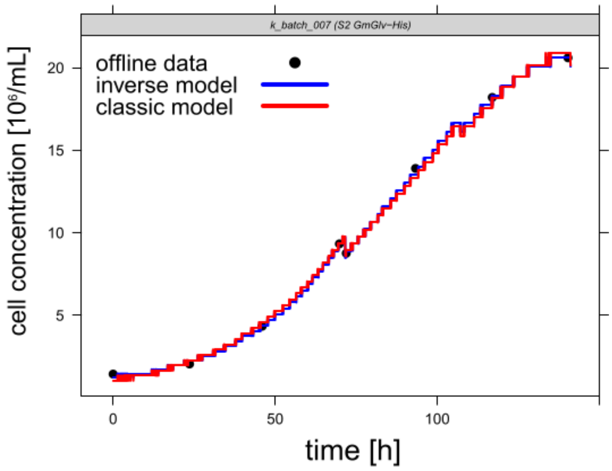

Figure S6. Offline cell concentration (black dot) and estimated time courses for cultivation k_batch_007 (inverse model in blue, classical model in red based on OD_{880}).

2.2 *Exploring a general realtionship using the full OD_{880} dataset*

2.2.1 *Regression using pooled OD_{880} data*

As a first attempt of establishing a generally valid model we pooled the data from all available cultivations and fitted standard regression models analog to the case for a single cultivation. Despite the fact that the fit represents the data appropriately, the other diagnostic plots show major problems, because residuals are neither normally distributed nor homoscedastic (Fig. S7). Consequently, this modelling approach does not describe the dataset adequately.

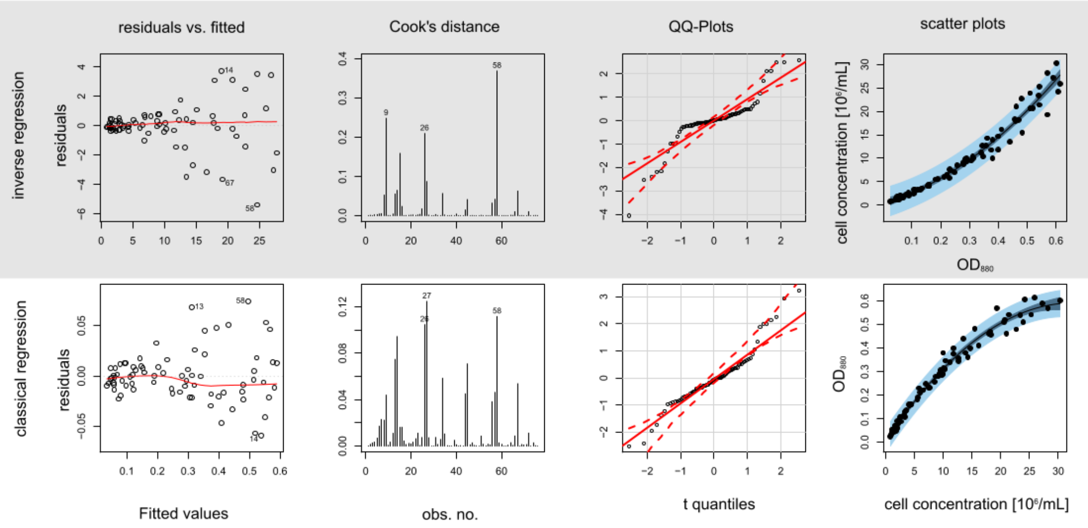

Figure S7. Diagnostic plots for inverse and classical regression for the full dataset with n=76: (a) residuals vs. fitted values for homoscedasticity evaluation, (b) Cook's distance plot for determination of points with high influence, (c) QQ-Plots for normality assessment and (d) scatter plots with pointwise confidence and prediction bands (level=0.95).

2.2.2 *Linear mixed effects models (LME) for inverse calibration based on the OD_{880} dataset*

The data used for the analysis were obtained from eleven experimental runs causing a hierarchical structure in our dataset, where each subgroup represents the time course of a single cultivation. This violates the independence assumption underlying linear regression, because responses from the same cultivation cannot be regarded as independent. To account for this data structure we employed linear mixed effects models (LME) with "cultivation run" as grouping factor [12]. Here the response is modeled as being composed of a systematic fixed effect (a common relationship between cell concentration and OD_{880}) and a random effect which accounts for an unknown deviation associated with each distinct experiment. Hence the cultivation runs are regarded as exemplary and each new run will result in an unknown individual deviation from the fixed effect. Variance, which is not explained by the fixed and random effects, is represented in the error $e_{i,j}$. In the case of inverse calibration this results in model Equations 10 and 11, where $cell\ conc_{i,j}$ is the j-th cell concentration of the i-th cultivation with its corresponding optical density $OD_{880_{i,j}}$. Fixed effects are denoted by β, whereas random effects are termed b_i. $\sigma_{b1}{}^2$,$\sigma_{b2}{}^2$ andσ_{b1b2} represent the corresponding (co)variance.

$$cell\ conc_{i,j} = \beta_{0\ inv} + (\beta_{1\ inv} + b_{1,i\ inv}) \cdot OD_{880_{i,j}} + (\beta_{2\ inv} + b_{2,i\ inv}) \cdot OD_{880_{i,j}}{}^2 + e_{i,j\ inv} \tag{10}$$

$$\begin{pmatrix} b_{1,i\ inv} \\ b_{2,i\ inv} \end{pmatrix} \sim \mathcal{N}_2\left(\begin{pmatrix} 0 \\ 0 \end{pmatrix}, \begin{pmatrix} \sigma_{b1}{}^2 & \sigma_{b1b2} \\ \sigma_{b1b2} & \sigma_{b2}{}^2 \end{pmatrix}\right) \tag{11}$$

To account for the observed heteroscedasticity the error $e_{i,j\ inv}$ was allowed to have an OD-dependent variance of the following form:

$$e_{i,j\ inv} \sim \mathcal{N}\left(0, \sigma^2 \cdot OD_{880_{i,j}}{}^{2\delta}\right) \tag{12}$$

Based on restricted maximum likelihood estimation (REML) the model was fit to the hierarchical dataset. The results are shown in R-output S8.

```
Linear mixed-effects model fit by REML
 Data: ODDatenRed
       AIC      BIC    logLik
  169.2678 187.5915 -76.63392

Random effects:
 Formula: ~(0 + OD + I(OD^2)) | cultivation_run
 Structure: General positive-definite, Log-Cholesky parametrization
         StdDev    Corr
OD        3.037481 OD
I(OD^2)  12.442086 -0.82
Residual  2.513469

Variance function:
 Structure: Power of variance covariate
 Formula: ~OD
 Parameter estimates:
    power
0.9491688

Fixed effects: CC ~ 1 + OD + I(OD^2)
               Value Std.Error DF   t-value p-value
(Intercept)  0.36456  0.076825 63  4.745325       0
OD          18.45652  1.634912 63 11.289001       0
I(OD^2)     42.40418  4.976843 63  8.520296       0

 Correlation:
        (Intr) OD
OD      -0.732
I(OD^2)  0.503 -0.852

Standardized Within-Group Residuals:
        Min          Q1         Med          Q3         Max
-3.82148533 -0.40507981 -0.04962088  0.42019529  2.51568090

Number of Observations: 76
Number of Groups: 11
```

Output S8. Linear mixed effects model (inverse approach): **Fixed effects** contains the $\hat{\beta}$ values; **Random effects** contains the estimated $\hat{\sigma}_{b1}$, $\hat{\sigma}_{b2}$, and $\hat{\sigma}$ values; **Variance function** contains the estimate for $\hat{\delta}$.

The pure fixed effects term, usable for population predictions, is represented in Equation 13.

$$cell\ conc_{i,j_{pred}} = 0.36456 + 18.45652 \cdot OD_{880_{i,j}} + 42.40418 \cdot OD_{880_{ij}}{}^{2} \quad (13)$$

Plotting the residuals of this model shows that the heteroscedasticity still remains (Fig. S9). However it is taken into consideration within the variance model (Equation 12) and hence the standardized residuals (defined as: $\hat{e}_{i,j\ inv} / (\hat{\sigma} \cdot OD_{880_{i,j}}{}^{\hat{\delta}})$ exhibit a uniform shape (Fig. S9).

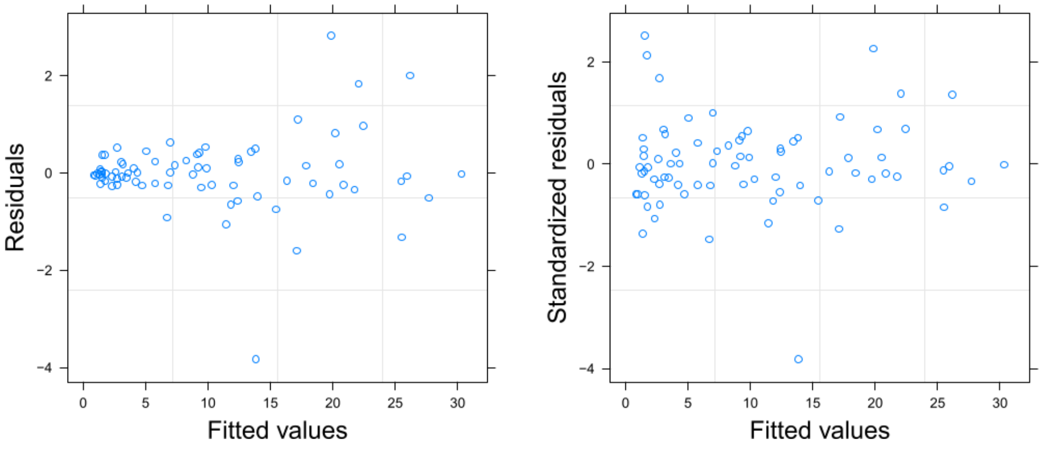

Figure S9. Residuals and standardized residuals of the linear mixed model (inverse case) plotted versus the fitted values.

The random effects estimators $\hat{b}_{1,i\ inv}$ and $\hat{b}_{2,i\ inv}$ are given for each group as follows (Output S10):

```
Random effects estimators:
                                        OD       I(OD^2)
k_fed_batch_005 (S2 GmGlv-GFP)    -2.74759893   9.5350569
k_batch_007 (S2 GmGlv-HisD7)      -0.64780345   1.4063047
k_batch_008 (S2 GmGlv-His D7)      1.65870522   1.5899946
k_batch_010 (S2 GmGlv-His D7)      3.10104030  -6.6422191
k_batch_013 (S2 GmGlv-His D7)     -0.80328069   2.3259866
k_batch_014 (S2 GmGlv His D7)     -2.22480018  15.3138551
k_fedbatch_011 (S2 GmGlv-His D7)  -0.09840578  -8.6924469
k_fedbatch_012 (S2 GmGlv-His D7)   4.30132184 -11.6169132
k_Perfusion 1 (S2 GmGlv-His D7)    2.23101414 -19.0722073
k_Perfusion_018 (S2 GmGlv His D7) -1.58894556   0.9727003
k_Perfusion_020 (S2 GmGlv His D7) -3.18124691  14.8798884
```

Output S10. Random effects estimators $\hat{b}_{1,i}$ and $\hat{b}_{2,i}$ (here indicated by OD and I(OD^2)).

Plotting the calibration curves for each subset shows that the fixed effects term describes the relationship adequately, especially for lower cell densities. Inter-cultivation variations (indicated by deviations of model predictions including the random effects terms (group predictions) from the model predictions of the purely fixed effects (population predictions)) become prevalent at higher cell densities (respectively at later cultivation stages, Fig. S11).

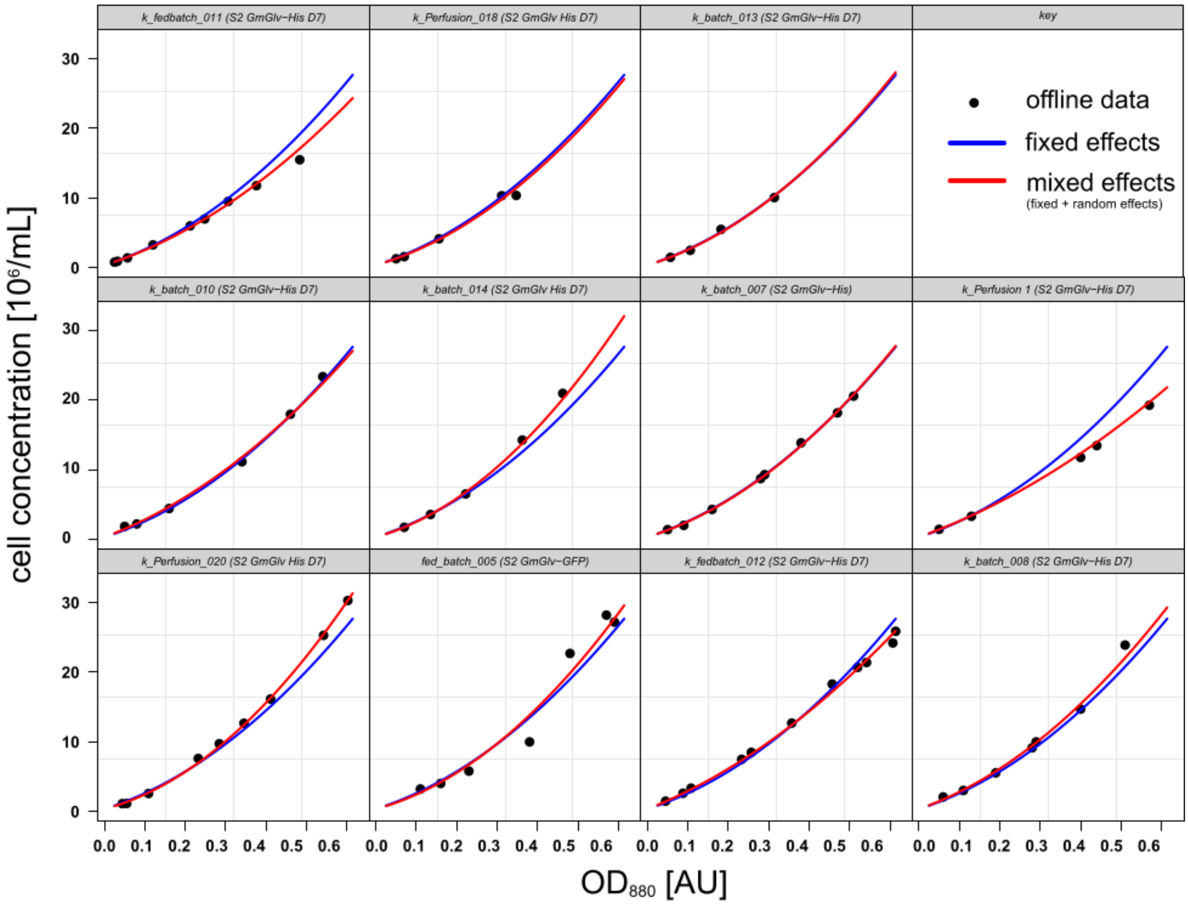

Figure S11. Scatter plots for the eleven cultivations: offline determined values are depicted as black dots, corresponding fixed effects and mixed effects predictions are shown as blue and red lines.

Based on the continuously measured sensor data, the fixed effects term (Equation 13) was used as a general method for cell density prediction (Fig. S12). Because the predicted growth curves are in good agreement with the offline data and represent plausible time courses, we assume that the fixed effects term can be used for online prediction in future experiments.

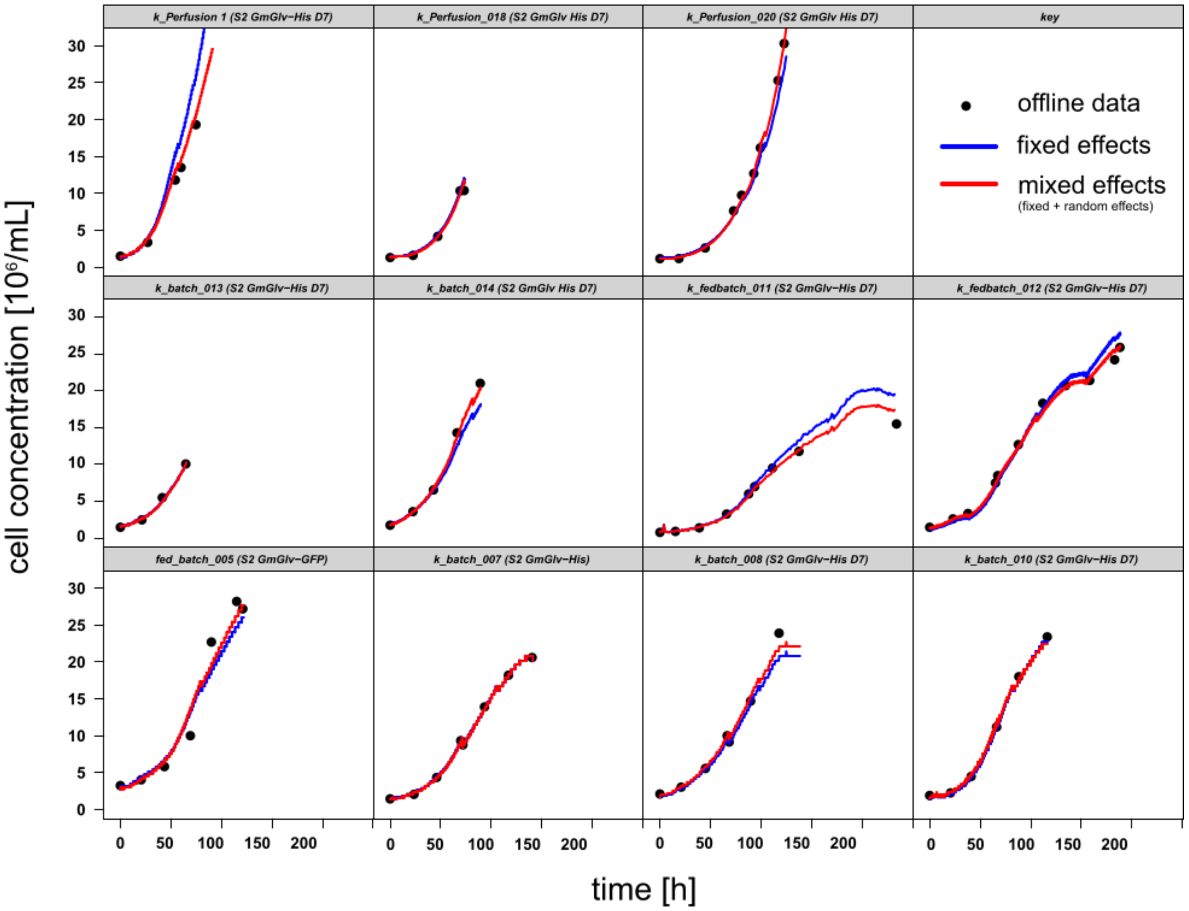

Figure S12. Time courses of cell concentration of eleven culture runs, based on the mixed effects predictions (red) and the pure fixed effects term (blue) (inverse calibration approach based on OD_{880}).

2.2.3 *Linear mixed effects models (LME) for classical calibration based on the OD_{880} dataset*

Analog to the inverse calibration an LME model was used for the classical approach. It is described by the following Equations:

$$OD_{880\,i,j} = \beta_{0\,cl} + \left(\beta_{1\,cl} + b_{1,i\,cl}\right) \cdot cell\ conc_{i,j} + \left(\beta_{2\,cl} + b_{2,i\,cl}\right) \cdot cell\ conc_{i,j}{}^{2} + e_{i,j\,cl} \qquad (14)$$

$$\begin{pmatrix} b_{1,i\,cl} \\ b_{2,i\,cl} \end{pmatrix} \sim \mathcal{N}_2\left(\begin{pmatrix} 0 \\ 0 \end{pmatrix}, \begin{pmatrix} \sigma_{b1}{}^{2} & \sigma_{b1b2} \\ \sigma_{b1b2} & \sigma_{b2}{}^{2} \end{pmatrix}\right) \qquad (15)$$

$$e_{i,j\,cl} \sim \mathcal{N}\left(0, \sigma^2 \cdot cell\ conc_{i,j}{}^{2\delta}\right) \qquad (16)$$

The model is summarized in R-output S13 and calibration curves as well as residual plots are shown in Figure S14.

```
Linear mixed-effects model fit by REML
 Data: ODDatenRed
        AIC       BIC   logLik
  -342.2721 -323.9485 179.1361

Random effects:
 Formula: ~(0 + CC + I(CC^2)) | cultivation_run
 Structure: General positive-definite, Log-Cholesky parametrization
         StdDev       Corr
CC       0.0024730272 CC
I(CC^2)  0.0001324089 -0.754
Residual 0.0071148947
```

```
Variance function:
 Structure: Power of variance covariate
 Formula: ~CC
 Parameter estimates:
    power
0.3754071

Fixed effects: OD ~ 1 + CC + I(CC^2)
                  Value   Std.Error DF   t-value p-value
(Intercept)  0.00318860 0.0026609945 63  1.198274  0.2353
CC           0.03684048 0.0011663508 63 31.586105  0.0000
I(CC^2)     -0.00057766 0.0000614199 63 -9.405026  0.0000

 Correlation:
        (Intr) CC
CC      -0.612
I(CC^2)  0.497 -0.857

Standardized Within-Group Residuals:
        Min          Q1         Med          Q3         Max
-2.82612284 -0.41949377  0.05622856  0.48276514  2.63196750

Number of Observations: 76

Number of Groups: 11


Random effects estimators:
                                            CC       I(CC^2)
k_fed_batch_005 (S2 GmGlv-GFP)     3.774719e-03 -1.602338e-04
k_batch_007 (S2 GmGlv-His)         1.306505e-04 -3.931560e-05
k_batch_008 (S2 GmGlv-His D7)     -1.322710e-03 -9.460547e-06
k_batch_010 (S2 GmGlv-His D7)     -1.169962e-03  3.141510e-05
k_batch_013 (S2 GmGlv-His D7)      3.490008e-04 -2.473990e-05
k_batch_014 (S2 GmGlv His D7)     -9.056331e-06 -1.226139e-04
k_fedbatch_011 (S2 GmGlv-His D7)   7.312492e-04  6.348296e-05
k_fedbatch_012 (S2 GmGlv-His D7)  -3.613356e-03  1.953135e-04
k_Perfusion 1 (S2 GmGlv-His D7)    1.971664e-03  5.706405e-05
k_Perfusion_018 (S2 GmGlv His D7)  1.266503e-03 -5.195154e-05
k_Perfusion_020 (S2 GmGlv His D7) -2.108702e-03  6.103968e-05
```

Output S13. Linear mixed effects model (classical approach): **Fixed effects** contains the $\hat{\beta}$ values; **Random effects** contains the estimated $\hat{\sigma}_{b1}$, $\hat{\sigma}_{b2}$ and $\hat{\sigma}$ values; **Variance function** contains the estimate for $\hat{\delta}$; **Random effects estimators** contains the estimated $\hat{b}_{1,i}$ and $\hat{b}_{2,i}$ (here indicated by CC and I(CC^2)).

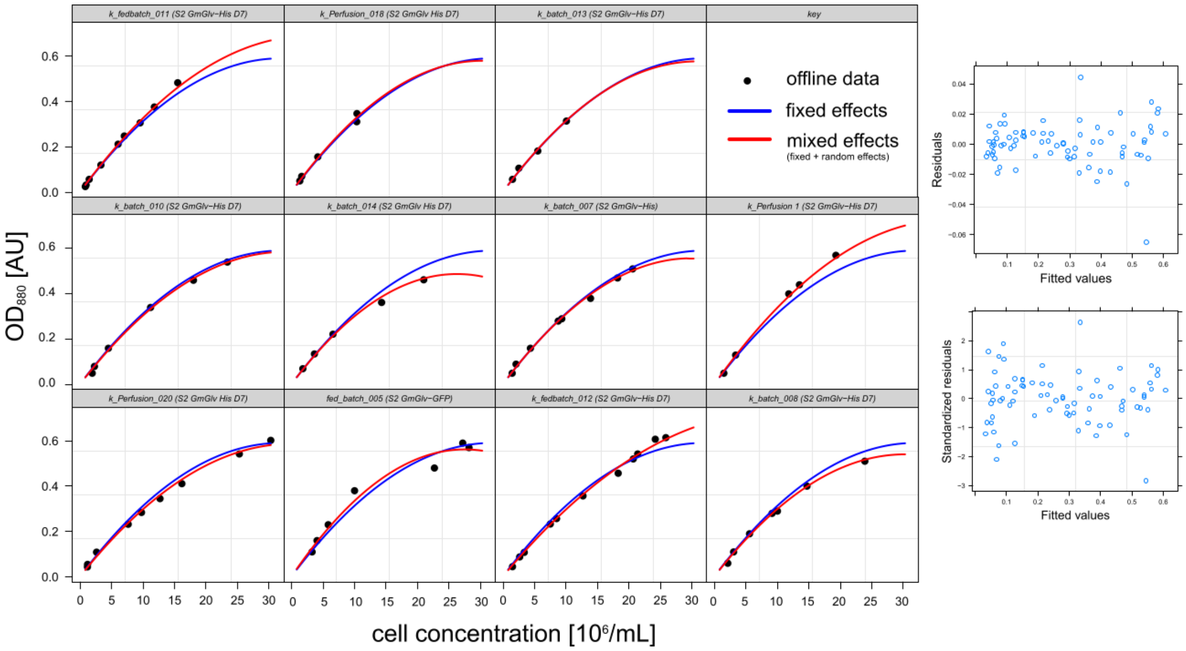

Figure S14. Scatter plots for eleven cultivations: offline determined values are depicted as black dots, corresponding fixed effects and mixed effects predictions are shown as blue and red lines (left panel). Plot of the raw and standardized residuals (right panel).

For prediction with the classical approach, the quadratic Equation 14 has to be rearranged according to the Equation 17.

$$cell\ conc_{i,j_{pred}} = \frac{-b + \sqrt{b^2 - 4ac}}{2a} \quad (17)$$

with

$$a = (\hat{\beta}_{2\ cl} + \hat{b}_{2,i\ cl})$$
$$b = (\hat{\beta}_{1\ cl} + \hat{b}_{1,i\ cl})$$
$$c = \left(\hat{\beta}_{0\ cl} - OD_{880_{i,j}}\right)$$

The pure fixed effects term (that may be used for future predictions) results from Equation 17 when $\hat{b}_{2,i\ cl} = \hat{b}_{1,i\ cl} = 0$. The resulting plots show nearly equal time courses compared to the inverse approach (Fig. S15).

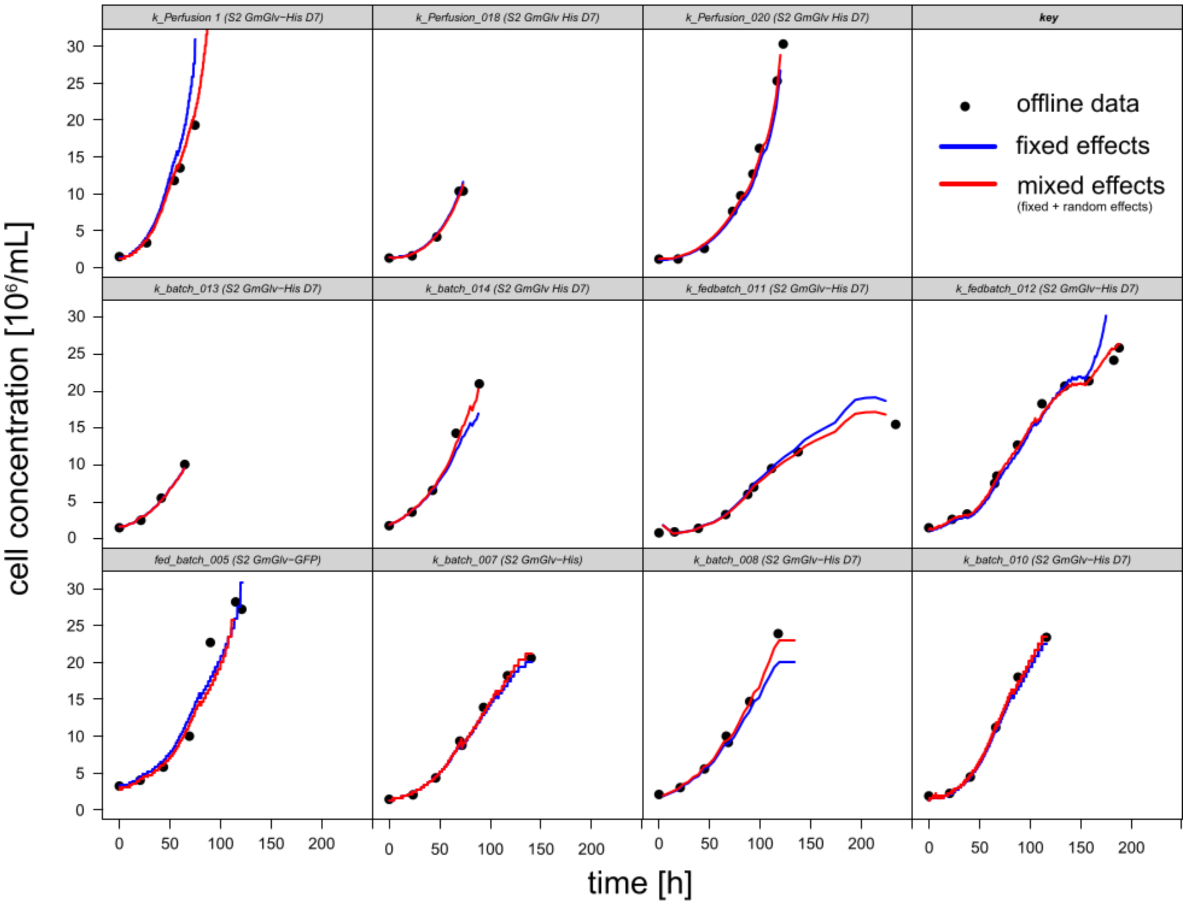

Figure S15. Time courses of cell concentration of eleven culture runs, based on the mixed effects predictions (red) and the pure fixed effects term (blue) (classical calibration approach based on OD_{880}).

3. Assessment of the permittivity ε for the prediction of the cell concentration

3.1 Selection of a suitable regressor variable

In contrast to the optical density measurement system the dielectric spectroscope provides five output variables (ε, Δε, f_c, α and conductivity), which are possible candidates for cell density prediction. In order to select a suitable variable and to reveal possible relationships within the group we constructed a scatter plot matrix with corresponding pairwise correlation coefficients (Fig. S16). Because three of the five variables (ε, Δε and α) show strong multicollinearity among each other, and since we wanted to keep the model simple, a multiple regression approach (utilizing several covariables) was considered inappropriate. We selected ε for further analysis as it shows the highest correlation with the cell concentration.

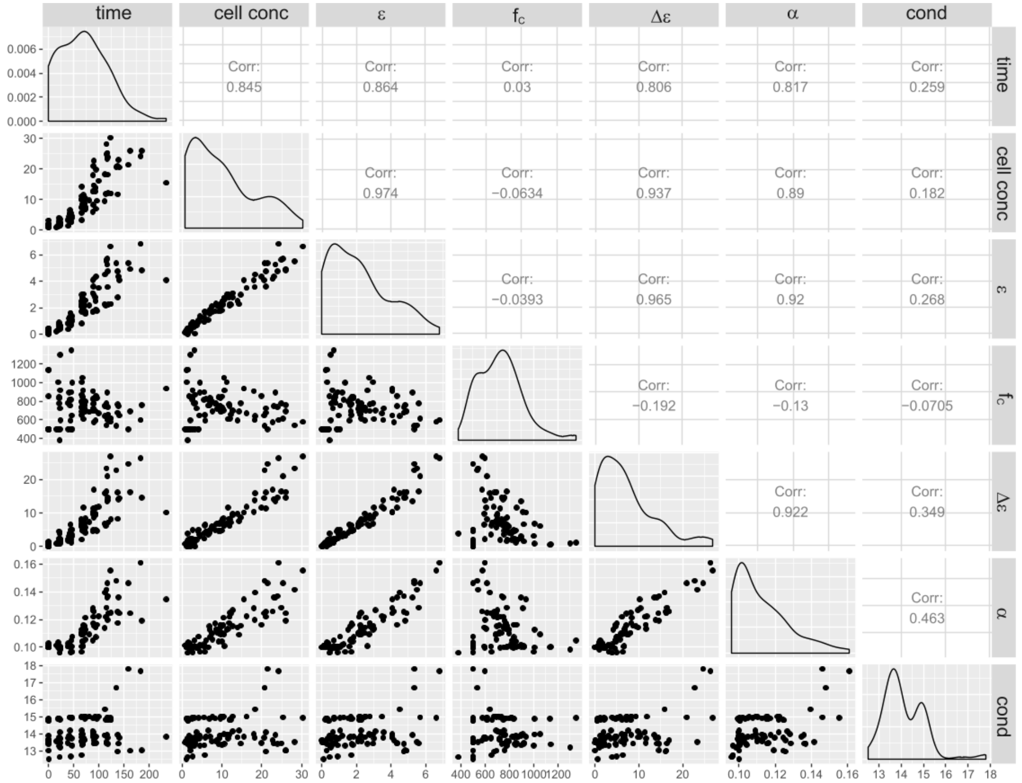

Figure S16. Scatter plot matrix for time, cell concentration and the corresponding output variables of the Incyte system.

3.2 Retrospective modeling with ordinary least squares (OLS) linear regression

The permittivity (ε) data of a single cultivation can be used for the reconstruction of a detailed growth curve. Based on an exemplary cultivation (k_batch_008) simple linear regression models were fitted to obtain the corresponding $\hat{\beta}$ values. Again classical and inverse approaches were considered (Equations 18 and 19).

$$cell\ conc = \beta_{0\ inv} + \beta_{1\ inv} \cdot \varepsilon + e_{inv} \qquad with\ \ e_{inv} \sim \mathcal{N}\left(0, {\sigma_{e_{inv}}}^2\right) \tag{18}$$

$$\varepsilon = \beta_{0\ cl} + \beta_{1\ cl} \cdot cell\ conc + e_{cl} \qquad with\ \ e_{cl} \sim \mathcal{N}\left(0, {\sigma_{e_{cl}}}^2\right) \tag{19}$$

Corresponding diagnositic plots and model outputs are shown below (Output S17, Fig. S18).

Inverse model
Coefficients:

	Estimate	Std. Error	t value	Pr(>|t|)	
$\hat{\beta}_{0\ inv}$	0.6166	0.3111	1.982	0.0947	.
$\hat{\beta}_{1\ inv}$	4.9803	0.1157	43.027	1.05e-08	***

Signif. codes: 0 ‘***’ 0.001 ‘**’ 0.01 ‘*’ 0.05 ‘.’ 0.1 ‘ ’ 1

Residual standard error: 0.5185 on 6 degrees of freedom
Multiple R-squared: 0.9968, Adjusted R-squared: 0.9962
F-statistic: 1851 on 1 and 6 DF, p-value: 1.055e-08

Classical model
Coefficients:

	Estimate	Std. Error	t value	Pr(>|t|)	
$\hat{\beta}_{0\ cl}$	-0.116394	0.064633	-1.801	0.122	
$\hat{\beta}_{1\ cl}$	0.200144	0.004652	43.027	1.05e-08	***

Signif. codes: 0 ‘***’ 0.001 ‘**’ 0.01 ‘*’ 0.05 ‘.’ 0.1 ‘ ’ 1

Residual standard error: 0.1039 on 6 degrees of freedom
Multiple R-squared: 0.9968, Adjusted R-squared: 0.9962
F-statistic: 1851 on 1 and 6 DF, p-value: 1.055e-08

Output S17. Inverse and classical regression models based on ε for cultivation k_batch_008.

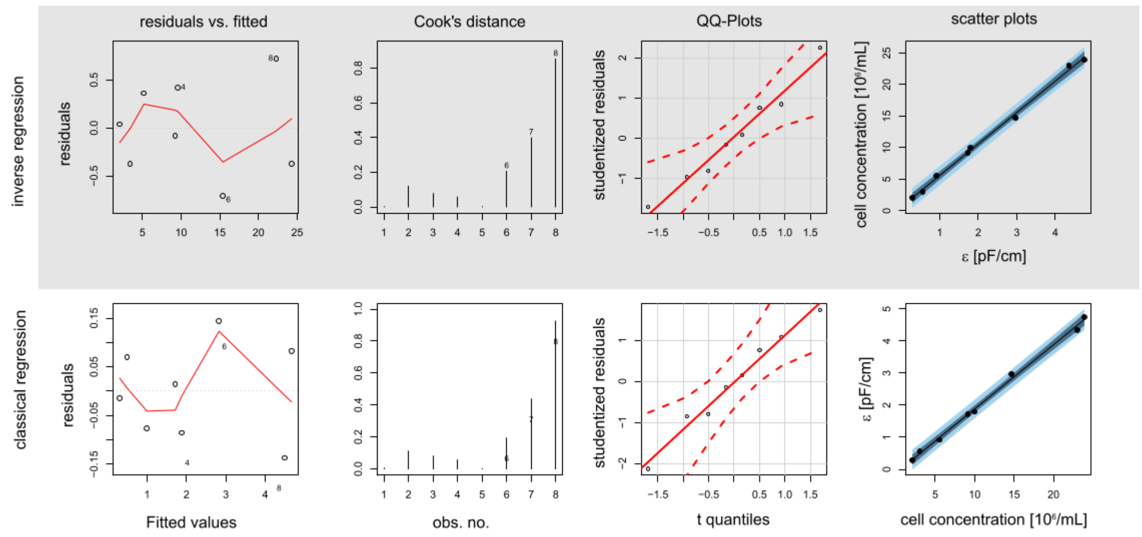

Figure S18. Diagnostic plots for inverse and classical regression: (a) Residuals vs. fitted values for homoscedasticity evaluation, (b) Cook's distance plot for determination of points with high influence, (c) QQ-Plots for normality assessment and (d) scatter plots with pointwise confidence and prediction bands (level=0.95).

Estimations based on both models yielded identical growth curves, as shown in Figure S19. This is again the result of a determination coefficient close to one (multiple and adj. $R^2 > 0.99$). In summary both methods, inverse and classical calibration, can be used for retrospective modelling based on ε.

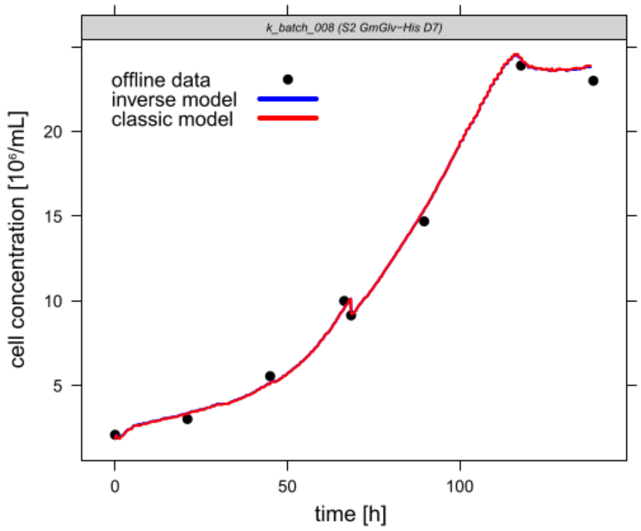

Figure S19. Time course of k_batch_008, prediction was based on the inverse and classical regression models with ε.

3.3 *Exploring a general realtionship using the full ε dataset*

3.3.1 *Regression using pooled ε data*

Similar to the OD_{880} case we pooled the ε data from all available cultivations and fitted standard regression models to get a first impression of the relationship between cell concentration and ε. Based on the scatter plot, it can be concluded that a linear term is sufficient, and the quadratic term from the OD_{880} model can be omitted. The overall fit represented the data sufficiently, but again residuals exhibited strong heteroscedasticity (Fig. S20).

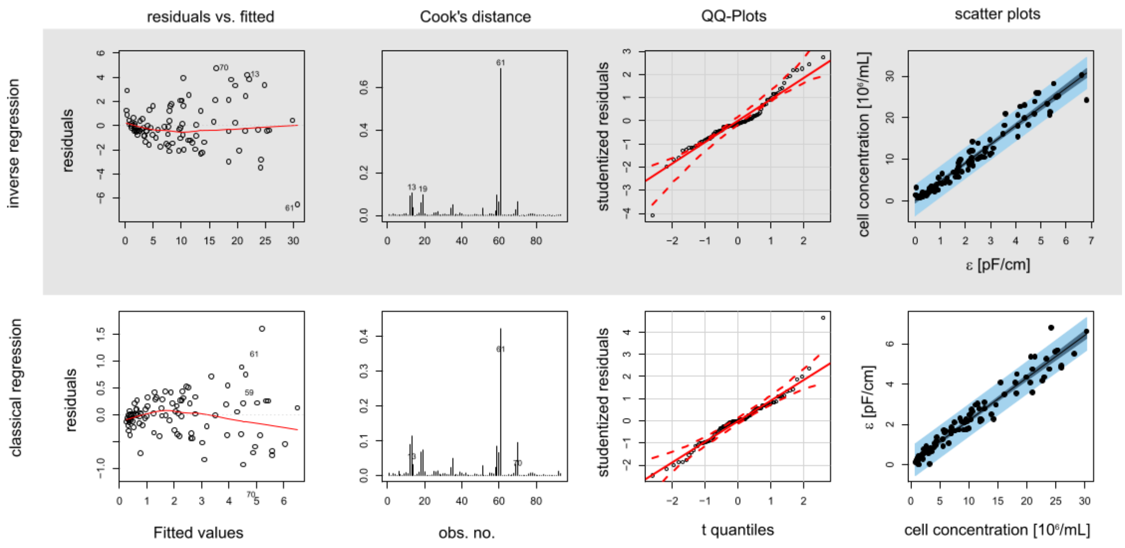

Figure S20. Diagnostic plots for inverse and classical regression of the pooled ε data: (a) Residuals vs. fitted values for homoscedasticity evaluation, (b) Cook's distance plot for determination of points with high influence, (c) QQ-Plots for normality assessment and (d) scatter plots with pointwise confidence and prediction bands (level=0.95).

3.3.2 Linear mixed effects models (LME) for inverse calibration based on the ε *data*

The full ε dataset consisted of thirteen subgroups, each representing the time course of a single cultivation. Based on the appearance of the scatter plots (Fig. S20) we decided to use a simple LME model according to Equations 20-22. Here, $cell\ conc_{i,j}$ indicates the j-th cell concentration of the i-th cultivation, $\beta_{0\ inv}$ and $\beta_{1\ inv}$ represent the fixed effects and $b_{1,i\ inv}$ denotes the cultivation dependent random effect with its variance ${\sigma_{b1}}^2$.

$$cell\ conc_{i,j} = \beta_{0\ inv} + \left(\beta_{1\ inv} + b_{1,i\ inv}\right) \cdot \varepsilon_{i,j} + e_{i,j\ inv} \qquad (20)$$

$$b_{1,i\ inv} \sim \mathcal{N}(0, {\sigma_{b1}}^2) \qquad (21)$$

During analysis it turned out that heteroscedasticity is not linked to the value of ε, but can be modeled using a constant, but cultivation-dependent variance (Equation 22). In the employed model δ_i is a parameter connecting the standard deviation of the residuals σ with a distinct cultivation i.

$$e_{i,j\ inv} \sim \mathcal{N}\left(0, \sigma^2 \cdot {\delta_i}^2\right) \qquad (22)$$

The corresponding model is shown in output S21.

```
Linear mixed-effects model fit by REML
 Data: ImpDatenRed
       AIC      BIC    logLik
  301.1257 341.2994 -134.5628

Random effects:
 Formula: ~0 + e | cultivation_run
                e Residual
StdDev: 0.5948544 2.032631

Variance function:
 Structure: Different standard deviations per stratum
 Formula: ~1 | cultivation_run
 Parameter estimates:
              k_batch_002(S2 GmGlv-GFP C9)           k_batch_004 (S2 GmGlv-GFP C9)
                                 1.0000000                               0.4895169
             k_batch_007 (S2 GmGlv-His D7)           k_batch_008 (S2 GmGlv-His D7)
                                 0.5935625                               0.3207547
```

```
            k_batch_010 (S2 GmGlv-His D7)             k_batch_013 (S2 GmGlv-His D7)
                                0.5021930                                 0.1503195
            k_batch_014 (S2 GmGlv His D7) k_batch_017 (S2 Mt-Glv-His AcGFPCoHygro A4)
                                0.3226619                                 0.4200694
         k_fedbatch_005 (S2 GmGlv-GFP C9)          k_fedbatch_011 (S2 GmGlv-His D7)
                                1.2036433                                 0.2508674
         k_fedbatch_012 (S2 GmGlv-His D7)         k_Perfusion_018 (S2 GmGlv-His D7)
                                0.4340070                                 0.2662578
        k_Perfusion_020 (S2 GmGlv-His D7)
                                0.1758531
Fixed effects: CC ~ 1 + e
                Value Std.Error DF   t-value p-value
(Intercept) -0.075501 0.1085423 79 -0.695595  0.4887
e            4.659552 0.1745077 79 26.701132  0.0000
 Correlation:
  (Intr)
e -0.217

Standardized Within-Group Residuals:
       Min         Q1        Med         Q3        Max
-2.2120705 -0.7987214  0.1535858  0.8277531  1.5350747

Number of Observations: 93
Number of Groups: 13

Random effects estimators:

                                                     e
k_batch_002(S2 GmGlv-GFP C9)                 0.2686939
k_batch_004 (S2 GmGlv-GFP C9)               -0.2321065
k_batch_007 (S2 GmGlv-His D7)                0.2982819
k_batch_008 (S2 GmGlv-His D7)                0.5179954
k_batch_010 (S2 GmGlv-His D7)               -0.3768779
k_batch_013 (S2 GmGlv-His D7)               -0.5258530
k_batch_014 (S2 GmGlv His D7)                1.2512570
k_batch_017 (S2 Mt-Glv-His AcGFPCoHygro A4)  0.4768638
k_fedbatch_005 (S2 GmGlv-GFP C9)             0.2396000
k_fedbatch_011 (S2 GmGlv-His D7)            -0.6765539
k_fedbatch_012 (S2 GmGlv-His D7)            -0.8083132
k_Perfusion_018 (S2 GmGlv-His D7)           -0.3214135
k_Perfusion_020 (S2 GmGlv-His D7)           -0.1115741
```

Output S21. Linear mixed effects model (inverse approach): **Fixed effects** contains the $\hat{\beta}$ values; **Random effects** contains the estimated $\hat{\sigma}_{b1}$ and $\hat{\sigma}$ values; **Variance function** contains the estimates for $\hat{\delta}_i$; **Random effects estimators** contains the estimated $\hat{b}_{1,i}$ values (here indicated by e).

The variance model was considered to be adequate, because the standardized residuals ($\hat{e}_{i,j\ inv}/(\hat{\sigma} \cdot \hat{\delta}_i)$) showed a uniform distribution (Fig. S22).

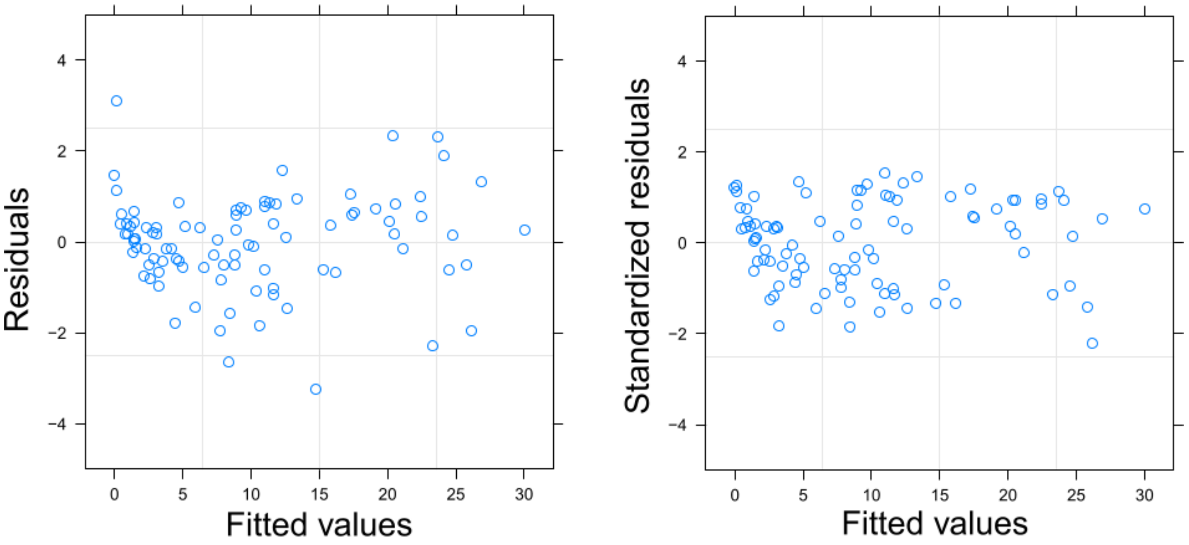

Figure S22. Residuals and standardized residuals of the linear mixed model (inverse case based on ε) plotted versus the fitted values.

Based on the fitted model, corresponding scatter plots were generated (Fig. S22). Growth curve estimations were based on the full time curses of ε using the fixed effects term (Equation 23, Fig. S23).

$$cell\ conc_{i,j_{pred}} =-\ 0.075501 +\ 4.659552 \cdot \varepsilon_{i,j} \tag{23}$$

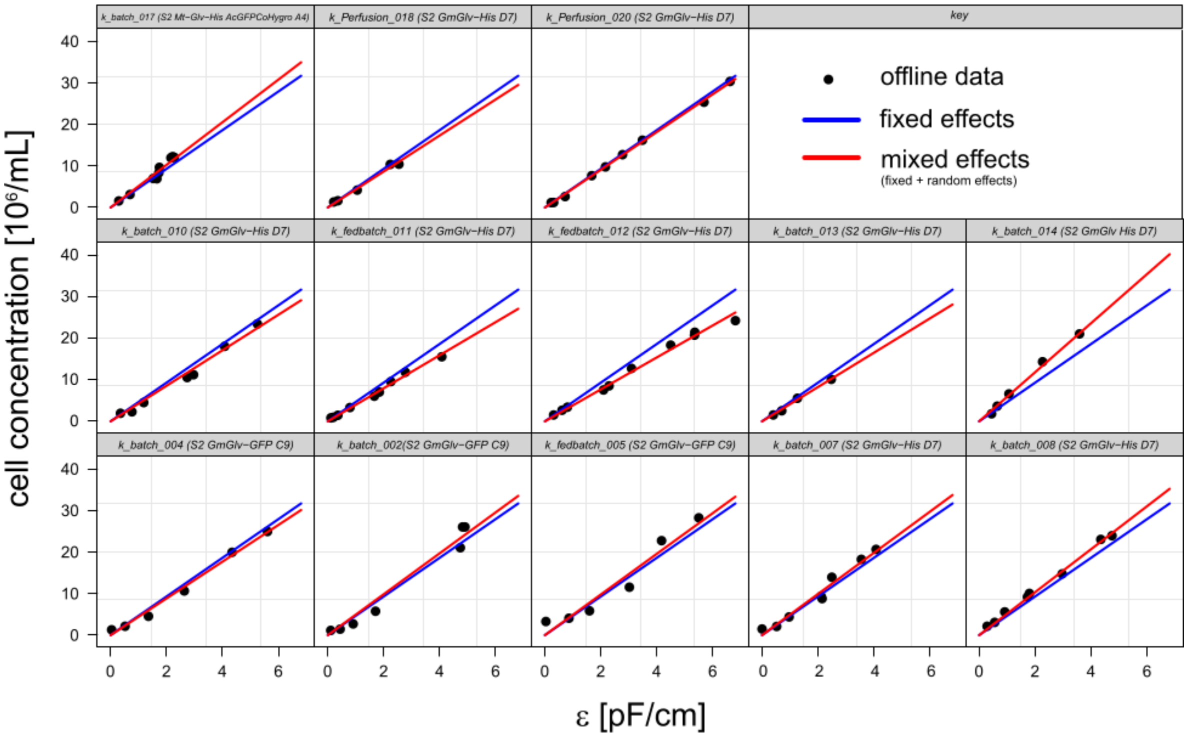

Figure S22. Scatter plots for thirteen cultivations: offline determined values are depicted as black dots, corresponding fixed effects and mixed effects predictions are shown as blue and red lines (inverse calibration approach based on ε).

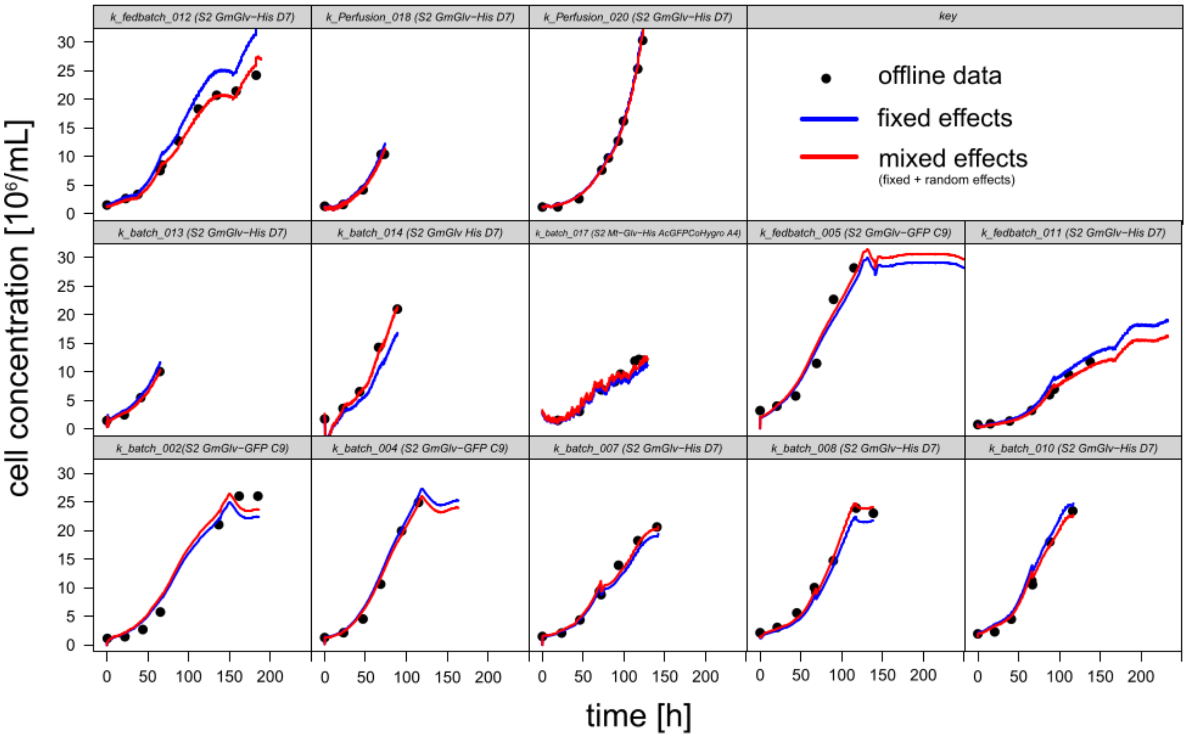

Figure S23. Time courses of cell concentration of thirteen culture runs, based on the mixed effects predictions (red) and the pure fixed term (blue) (inverse calibration approach based on ε).

3.3.3 Linear mixed effects models (LME) for classical calibration based on the ε data

Classical calibration using a LME model is described by Equations 24-26.

$$\varepsilon_{i,j} = \beta_{0\,cl} + \left(\beta_{1\,cl} + b_{1,i\,cl}\right) \cdot cell\ conc_{i,j} + e_{i,j\,cl} \tag{24}$$

$$b_{1,i\,cl} \sim \mathcal{N}(0, {\sigma_{b1}}^2) \tag{25}$$

$$e_{i,j\,cl} \sim \mathcal{N}\left(0, \sigma^2 \cdot {\delta_i}^2\right) \tag{26}$$

The model is summarized in Output S24 and residual plots as well as calibration curves are shown in Figure S25 and S26.

```
Linear mixed-effects model fit by REML
 Data: ImpDatenRed
       AIC      BIC   logLik
  24.09597 64.26973 3.952013

Random effects:
 Formula: ~0 + CC | cultivation_run
                CC  Residual
StdDev: 0.02696844 0.3984836

Variance function:
 Structure: Different standard deviations per stratum
 Formula: ~1 | cultivation_run
 Parameter estimates:
              k_batch_002(S2 GmGlv-GFP C9)            k_batch_004 (S2 GmGlv-GFP C9)
                                   1.0000000                              0.5568557
             k_batch_007 (S2 GmGlv-His D7)            k_batch_008 (S2 GmGlv-His D7)
                                   0.6009229                              0.3409433
             k_batch_010 (S2 GmGlv-His D7)            k_batch_013 (S2 GmGlv-His D7)
                                   0.5791894                              0.1758280
             k_batch_014 (S2 GmGlv His D7) k_batch_017 (S2 Mt-Glv-His AcGFPCoHygro A4)
                                   0.2582992                              0.3995654
          k_fedbatch_005 (S2 GmGlv-GFP C9)         k_fedbatch_011 (S2 GmGlv-His D7)
                                   1.2257886                              0.3306219
          k_fedbatch_012 (S2 GmGlv-His D7)         k_Perfusion_018 (S2 GmGlv-His D7)
                                   0.5843147                              0.3194630
         k_Perfusion_020 (S2 GmGlv-His D7)
                                   0.1907367
Fixed effects: e ~ 1 + CC
                 Value   Std.Error DF  t-value p-value
(Intercept) 0.03968056 0.023447573 79  1.69231  0.0945
CC          0.21435331 0.007907207 79 27.10860  0.0000
 Correlation:
   (Intr)
CC -0.223

Standardized Within-Group Residuals:
       Min         Q1        Med         Q3        Max
-1.3678591 -0.7751521 -0.2054960  0.8244499  2.3656666

Number of Observations: 93
Number of Groups: 13

Random effects estimators:
                                                      CC
k_batch_002(S2 GmGlv-GFP C9)                -0.015264209
k_batch_004 (S2 GmGlv-GFP C9)                0.009308196
k_batch_007 (S2 GmGlv-His D7)               -0.016119982
k_batch_008 (S2 GmGlv-His D7)               -0.023070406
k_batch_010 (S2 GmGlv-His D7)                0.016353649
k_batch_013 (S2 GmGlv-His D7)                0.023914405
k_batch_014 (S2 GmGlv His D7)               -0.048541393
k_batch_017 (S2 Mt-Glv-His AcGFPCoHygro A4) -0.024068530
k_fedbatch_005 (S2 GmGlv-GFP C9)            -0.015074243
k_fedbatch_011 (S2 GmGlv-His D7)             0.033262676
k_fedbatch_012 (S2 GmGlv-His D7)             0.042650736
k_Perfusion_018 (S2 GmGlv-His D7)            0.012375035
k_Perfusion_020 (S2 GmGlv-His D7)            0.004274067
```

Output S24. Linear mixed-effects model (classical approach): **Fixed effects** contains the $\hat{\beta}$ values; **Random effects** contains the estimated $\hat{\sigma}_{b1}$ and $\hat{\sigma}$ values; **Variance function** contains the estimates for $\hat{\delta}_i$; **Random effects estimators** contains the estimated $\hat{b}_{1,i}$ values (here indicated by CC).

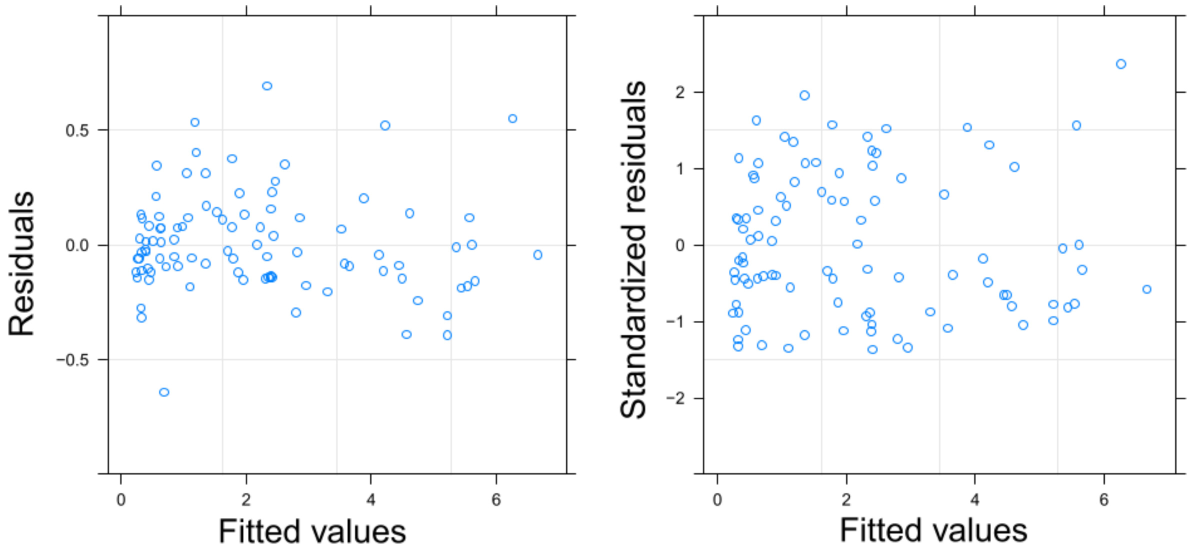

Figure S25. Residuals and standardized residuals of the linear mixed model (classical case based on ε) plotted versus the fitted values.

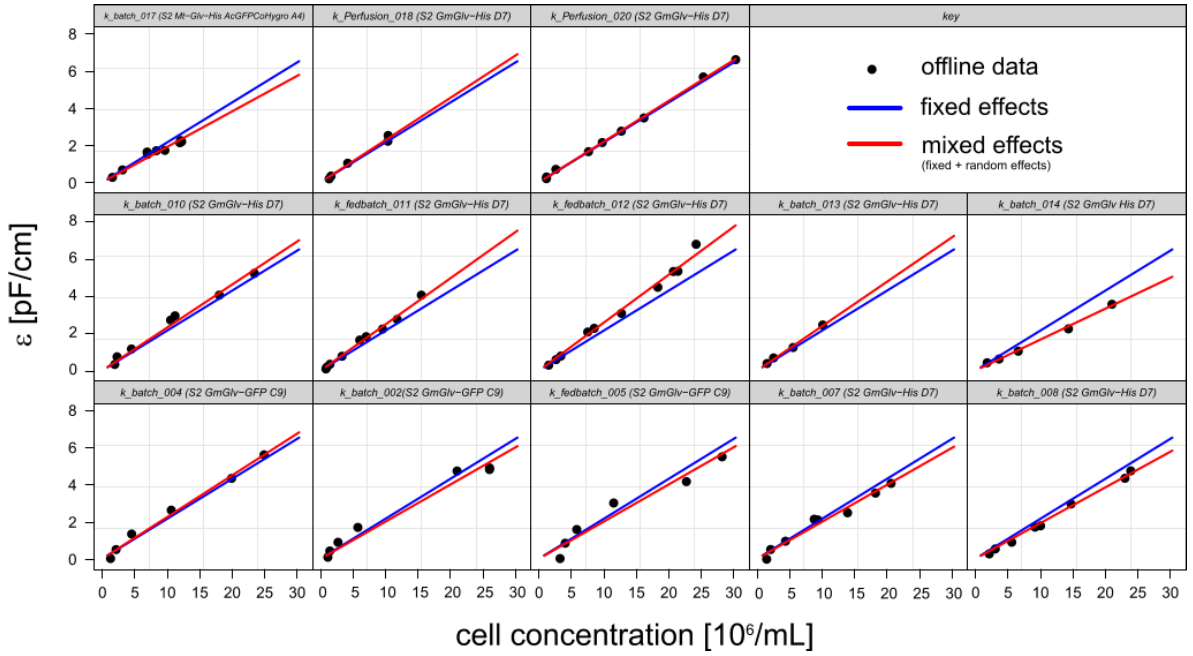

Figure S26. Scatter plots for thirteen cultivations: offline determined values are depicted as black dots, corresponding fixed effects and mixed effects predictions are shown as blue and red lines (classical case based on ε).

Prediction is enabled by rearranging the model according to Equation 27 (Fig. S27).

$$cell\ conc_{i,j_{pred}} = \frac{\varepsilon_{i,j} - \hat{\beta}_{0\ cl}}{\hat{\beta}_{1\ cl} + \hat{b}_{1,i\ cl}} \tag{27}$$

For population predictions only the fixed effects model is used, hence $\hat{b}_{1,i\ cl} = 0$.

$$cell\ conc_{i,j_{pred}} = \frac{\varepsilon_{i,j} - \hat{\beta}_{0\ cl}}{\hat{\beta}_{1\ cl}} = \frac{\varepsilon_{i,j} - 0.03968056}{0.21435331} \tag{28}$$

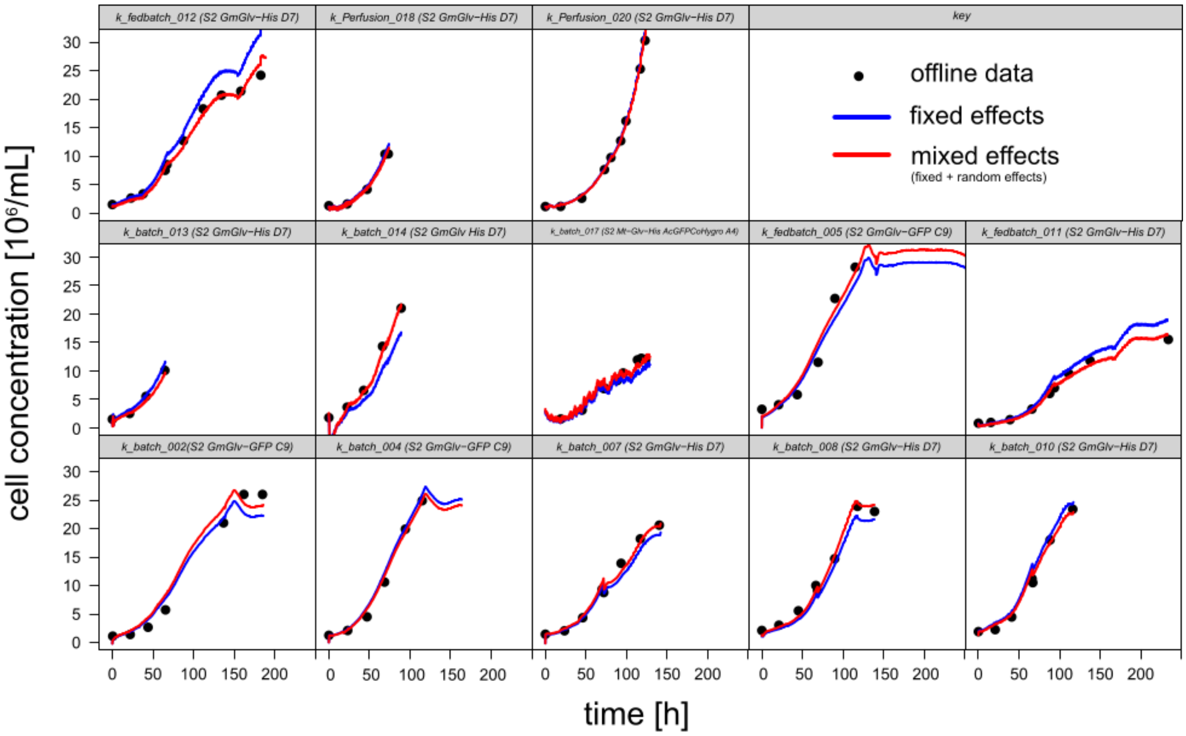

Figure S27. Time courses of cell concentration of thirteen culture runs, based on the mixed effects predictions (red) and the pure fixed effects term (blue) (classical calibration approach based on ε).

4. Summary

Within this analysis we investigated the relationship between cell concentration and optical density (OD_{880}) or permittivity (ε) for hierarchical datasets originating from different cultivations. Thereby the employment of LME models accounted for the grouping structure and heteroscedasticity. Concerning the assignment of the dependent and independent variable, inverse and classical approaches were tested, yielding corresponding models for cell density prediction. Because we were able to predict plausible time courses using only the fixed effects terms, it was assumed that the fixed effects terms are also applicable for future predictions (Table S28). From a practical viewpoint all models yielded comparable results. However, in a strict mathematical sense, only the classical models are in agreement with the assumption, that an *error free cell concentration* causes a *stochastic signal output*.

Table S28. Summary of the fixed effects Equation for cell concentration prediction

Prediction based on optical density OD_{880}	
Inverse calibration	$cell\ conc_{predicted,\,inv} = 0.36456 + 18.45652 \cdot OD_{880} + 42.40418 \cdot OD_{880}{}^{2}$
Classical calibration	$cell\ conc_{predicted,\,cl} = \dfrac{-0.03684048 + \sqrt{0.03684048^2 - (-4 \cdot 0.00057766 \cdot (0.00318860 - OD_{880}))}}{-2 \cdot 0.00057766}$
**Prediction based on permittivity ε**	
Inverse calibration	$cell\ conc_{predicted,\,inv} = -\,0.075501 + 4.659552 \cdot \varepsilon$
Classical calibration	$cell\ conc_{predicted,\,cl} = \dfrac{\varepsilon - 0.03968056}{0.21435331}$

To account for measurement error in the dependent and independent variable different error-in-variable-models can be used, including Deming regression or Passing-Bablok regression. However, these methods are not capable of representing the grouping structure of our data and were consequently not used in this analysis.

References

1. Besalú, E. The connection between inverse and classical calibration. *Talanta* **2013**, *116*, 45–49, doi:10.1016/j.talanta.2013.04.054.
2. Centner, V.; Massart, D. L.; Jong, S. de Inverse calibration predicts better than classical calibration. *Fresenius J. Anal. Chem.* **1998**, *361*, 2–9, doi:10.1007/s002160050825.
3. Krutchkoff, R. G. Classical and Inverse Regression Methods of Calibration. *Technometrics* **1967**, *9*, 425–439, doi:10.1080/00401706.1967.10490486.
4. R. Core Team *R: A language and environment for statistical computing. R Foundation for Statistical Computing*; Vienna, Austria, 2017; ISBN 3-900051-07-0.
5. Hadley Wickham; Hester, J.; Francois, R. *readr: Read Rectangular Text Data*; 2017;
6. Fox, J.; Weisberg, S. *An R companion to applied regression*; Sage Publications, 2011;
7. Greenwell, B. M.; Schubert Kabban, C. M. investr: an R package for inverse estimation. *R J.* **2014**, *6*, 90–100.
8. Barret Schloerke; Jason Crowley; Di Cook; Francois Briatte; Moritz Marbach; Edwin Thoen; Amos Elberg; Joseph Larmarange *GGally: Extension to "ggplot2"*; 2017;
9. Sarkar, D. *Lattice: multivariate data visualization with R*; Use R!; Springer: New York, 2008; ISBN 978-0-387-75968-5.
10. Deepayan Sarkar; Felix Andrews *latticeExtra: Extra Graphical Utilities Based on Lattice*; 2016;
11. Jose Pinheiro; Douglas Bates; Saikat DebRoy; Deepayan Sarkar *Linear and Nonlinear Mixed Effects Models*; 2017;
12. Pinheiro, J.; Bates, D. M. *Mixed-Effects Models in S and S-PLUS*; 1st ed.; Springer: New York, 2001;

© 2018 by the authors. Licensee MDPI, Basel, Switzerland. This article is an open access article distributed under the terms and conditions of the Creative Commons Attribution (CC BY) license (http://creativecommons.org/licenses/by/4.0/).
