# Supplementary material for: Dielectric Spectroscopy and Optical Density Measurement for the Online Monitoring and Control of Recombinant Protein Production in Stably Transformed Drosophila melanogaster S2 Cells
Source: Sensors (Basel). 2018 Mar 18;18(3):900. doi: 10.3390/s18030900 (PMC5876727; doi:10.3390/s18030900)

sensors

MDPI

Supplementary material S2

Raw values of the measured parameters in the small-scale viability assay

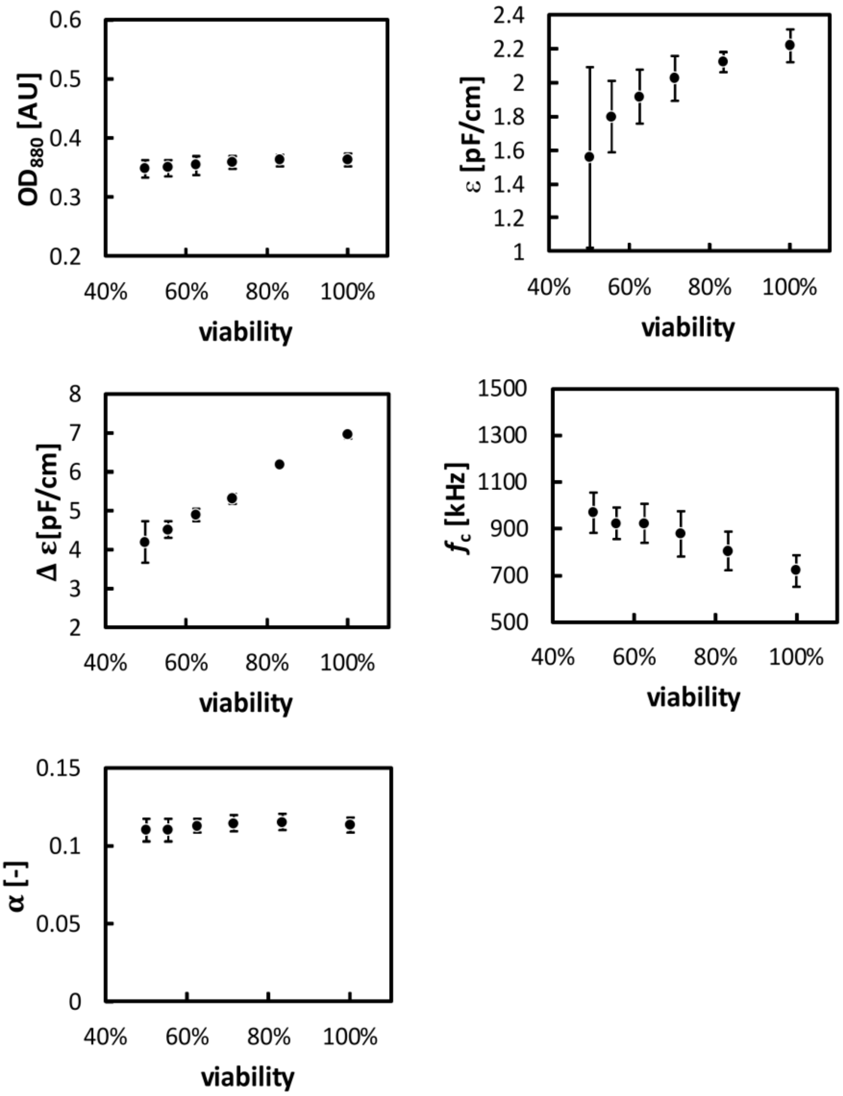

Supplement: Supplementary file 1 [file sensors-18-00900-s001.zip › Supplementary S2.pdf]
